# Site-Dependent Relationships Between Fungal Community Composition, Plant Genotypic Diversity and Environmental Drivers in a Salix Biomass System

**DOI:** 10.3389/ffunb.2021.671270

**Published:** 2021-08-13

**Authors:** Stefanie Hoeber, Christel Baum, Martin Weih, Stefano Manzoni, Petra Fransson

**Affiliations:** ^1^Department of Crop Production Ecology, Swedish University of Agricultural Sciences, Uppsala, Sweden; ^2^Soil Science, Faculty of Agricultural and Environmental Sciences, University of Rostock, Rostock, Germany; ^3^Department of Physical Geography and Bolin Centre for Climate Research, Stockholm University, Stockholm, Sweden; ^4^Department of Forest Mycology and Plant Pathology, Uppsala BioCenter, Swedish University of Agricultural Sciences, Uppsala, Sweden

**Keywords:** plant diversity, soil fungal community, ITS2, ectomycorrhizal fungi, *Salix*, short rotation coppice, plant genotypic variation

## Abstract

Soil fungi are strongly affected by plant species or genotypes since plants modify their surrounding environment, but the effects of plant genotype diversity on fungal diversity and function have not been extensively studied. The interactive responses of fungal community composition to plant genotypic diversity and environmental drivers were investigated in *Salix* biomass systems, posing questions about: (1) How fungal diversity varies as a function of plant genotype diversity; (2) If plant genotype identity is a strong driver of fungal community composition also in plant mixtures; (3) How the fungal communities change through time (seasonally and interannually)?; and (4) Will the proportion of ECM fungi increase over the rotation? Soil samples were collected over 4 years, starting preplanting from two *Salix* field trials, including four genotypes with contrasting phenology and functional traits, and genotypes were grown in all possible combinations (four genotypes in Uppsala, Sweden, two in Rostock, Germany). Fungal communities were identified, using Pacific Biosciences sequencing of fungal ITS2 amplicons. We found some site-dependent relationships between fungal community composition and genotype or diversity level, and site accounted for the largest part of the variation in fungal community composition. Rostock had a more homogenous community structure, with significant effects of genotype, diversity level, and the presence of one genotype (“Loden”) on fungal community composition. Soil properties and plant and litter traits contributed to explaining the variation in fungal species composition. The within-season variation in composition was of a similar magnitude to the year-to-year variation. The proportion of ECM fungi increased over time irrespective of plant genotype diversity, and, in Uppsala, the 4-mixture showed a weaker response than other combinations. Species richness was generally higher in Uppsala compared with that in Rostock and increased over time, but did not increase with plant genotype diversity. This significant site-specificity underlines the need for consideration of diverse sites to draw general conclusions of temporal variations and functioning of fungal communities. A significant increase in ECM colonization of soil under the pioneer tree *Salix* on agricultural soils was evident and points to changed litter decomposition and soil carbon dynamics during *Salix* growth.

## Introduction

Fungi are principal drivers of biogeochemical cycling, linking above- and belowground ecosystem components through their roles as symbiotic mediators of plant nutrient uptake (Smith and Read, 2008), and as decomposers of litter (Schneider et al., 2012). Soil fungal communities are strongly influenced by plant genotypes or species (Prescott and Grayston, 2013; Gallart et al., 2018b), since plants modify their surrounding environment (De Deyn et al., 2008), for example by providing living and dead organic matter, and by feeding soil microbial communities *via* rhizodeposition (Steinauer et al., 2016). All these plant-derived substrates are likely to be affected by plant genotypes, species, and diversity (Weih and Nordh, 2002; Hoeber et al., 2020). The relationships between plant diversity and ecosystem functions are complex (Gamfeldt et al., 2013; van der Plas et al., 2016; Baeten et al., 2019), but because of the interactions between fungi and plants, these relationships are likely mediated and possibly explained by the composition and function of soil fungi. In this contribution, we explore the links between fungal communities and plant genotype diversity.

Most studies of plant genotypes impact on fungal communities have focused on differences between individual genotypes, and very few on genotype mixture or diversity effects (e.g., Baum et al., 2018). Host genotypes may be crucial to structuring ectomycorrhizal (ECM) fungal communities, both compositional (Püttsepp et al., 2004; Korkama et al., 2006; Hrynkiewicz et al., 2010; Velmala et al., 2013; Gehring et al., 2017) and structural (Lang et al., 2013), and may affect the ability to form mycorrhiza (Tagu et al., 2005; Gherghel et al., 2014). However, no support for host genotype structuring ECM assemblages has been reported (Bubner et al., 2013; Lang et al., 2013). A plant genotype can also affect other non-symbiotic root-associated fungal communities (Baum et al., 2018; Gallart et al., 2018b; Bonito et al., 2019). Many previous studies were conducted on seedlings or younger trees; thus, the extent to which variation in tree genetics influences the structure and function of fungal communities within populations of adult trees remains an open question.

In addition to the fungal compositional changes, a plant genotype also affects the functional outcomes of the observed community shifts. Utilizing natural genetic variation in *Populus* hybrid populations, Schweitzer et al. (2011) tested whether stand gene diversity structures soil microbial communities, finding correlations between tree gene diversity, plant secondary chemistry and microbial community composition, impacting soil nitrogen availability. These results demonstrated that the effects of plant genetic diversity on other organisms may be mediated by the variation in plant functional traits (Schweitzer et al., 2011). In another study, rhizosphere and root-associated fungal communities in *P. radiata* differed in their response to both tree genotype and organic or inorganic nitrogen additions, suggesting that microbial communities more closely associated with roots were more sensitive to genotype-specific responses (Gallart et al., 2018a). Furthermore, fine root growth and soil and root-associated fungal abundance and activity changed under host genotype mixtures caused by changed competitive conditions for individual plant genotypes (Elferjani et al., 2014; Baum et al., 2018). Since soil fungal communities are central to plant nutrient supply, plant biomass production, and litter decomposition, they can significantly affect the carbon (C) storage in plant biomass and soil organic matter. Therefore, analyses of the impact of plant genotype diversity on soil fungal diversity can contribute to develop a rationale for the definition of genotype-specific optimization of tree diversity to combine high biomass productivity with high total C storage.

Tree species or genotype identity can sometimes have stronger impacts on ecosystem processes and organisms than diversity *per se* (Scherer-Lorenzen et al., 2005). The presence of a given genotype with effects on fungal communities can thus determine soil fungal community responses to genotype mixtures at different diversity levels. However, there are also studies that have shown no or little effect of host identity on fungal communities associated with willows (Erlandson et al., 2016, 2018; Arraiano-Castilho et al., 2020). Apart from genotype, fungal communities are strongly driven by environmental factors, including those changing during the growing season; especially soil pH and nutrient status (Lauber et al., 2008; Tedersoo et al., 2014). Several studies have shown that, for a given plant species, soil properties have a stronger effect on the soil and root-associated fungal community composition than the host genotype (Karlinski et al., 2013; Cregger et al., 2018; Bonito et al., 2019). Additionally, temporal trends in fungal community composition in relation to plant host genotype occur, both between and within seasons. Although less is known about seasonal variation, seasonal differences in the composition of, e.g., saprotrophic microfungi associated with roots of two *Salix* clones were observed previously by Baum and Hrynkiewicz (2006). Due to the dependence of soil fungal communities on climatic and edaphic factors, as well as plant community composition and/or the tree host, assessment of the interactive responses to biotic and abiotic factors has been put forward as a major challenge in fungal ecology (van der Heijden et al., 2015).

We addressed this challenge, using fast-growing *Salix* genotypes as a case study. *Salix* genotypes are cultivated in a short rotation coppice to produce biomass for energy purposes (Weih, 2004), and the cultivation of *Salix* is considered a sustainable biomass source with a positive greenhouse gas balance (Cunniff et al., 2015). Instead of planting single *Salix* genotypes, increased plant diversity has been proposed to be advantageous in terms of decreased pest, disease, and abiotic stress, and increased plant productivity and nutrient status (McCracken et al., 2011). Due to the fast growth and great variability among genotypes or closely related species with different plant traits, *Salix* is a suitable woody perennial model system to test for effects of both genotype and environmental factors on different ecosystem processes (Weih et al., 2019). *Salix* is a dual mycorrhizal plant genus, forming ECM and arbuscular mycorrhizal (AM) associations, with the two types of fungi colonizing roots with an antagonistic behavior (Lodge and Wentworth, 1990; Baum et al., 2018). In northern Europe, species of *Salix* have been shown to predominantly form ECM associations (Püttsepp et al., 2004). We used a short-rotation coppice system, where four genotypes of *Salix* were grown in all possible combinations, offering an opportunity to study the effect of plant genotypic diversity on soil fungal community composition. Previous studies from those trials showed genotype identity to have a more important influence than genotype diversity on shoot biomass productivity (Hoeber et al., 2018), and leaf litter quality coupled to genotype drive litter decomposition more than stand diversity or climate (Hoeber et al., 2020). The trials were established on former arable land (Hoeber et al., 2018) where we expect ECM fungal colonization to be sparse at the establishment and increase over time (see Kalucka and Jagodzinski, 2016). The aim of the present study was to describe the soil fungal communities, posing the following research questions with a focus on the potential drivers of soil fungal community structure and diversity: (1) How does fungal diversity vary as a function of plant diversity; (2) Is plant genotype identity a strong driver of fungal community composition even in tree mixtures?; (3) How does the fungal community change through time (seasonally and interannually)?; and (4) Will the proportion of ECM fungi within the total fungal community increase over the rotation, from the establishment on arable land and up until the first biomass harvest? Finally, the significance of plant genotype diversity on the soil fungal community composition was tested in relation to other drivers (plant biomass, litter chemistry, litter decomposition, soil properties, fungal biomass).

## Materials and Methods

### Study Sites

Two field trials were established on arable land in May, 2014, in Uppsala, Sweden (59°49' N 17°39' E) and Rostock, Northern Germany (54°02' N 12°05' E) within the ECOLINK-Salix trial (Hoeber et al., 2018), which is part of the TreeDivNet global tree diversity network (Verheyen et al., 2016). Four genotypes of *Salix*, partly differing at species or intraspecific levels, were used as the stand components: “Björn” (*Salix schwerinii* E. Wolf. × *S. viminalis* L.), “Jorr” (*S. viminalis*), “Loden” (*S. dasyclados*Wimm.), and “Tora” (*S. schwerinii* × *S. viminalis*). “Björn” and “Tora” are taxonomically closely related as they derive from the same parent material and are siblings but differ in some morphological and functional characteristics. “Jorr” is more closely related to the siblings “Björn” and “Tora,” whereas “Loden” belongs to a different species and thus is taxonomically most distant from the other three varieties. The genotypes were planted in pure cultures and various mixtures (2-, 3-, and 4-mixtures, giving four different plant diversity levels) and arranged in a randomized block design with a total of 45 plots over three blocks in Uppsala (15 plots per block). In Rostock, only the two genotypes “Loden” and “Tora” were grown, resulting in a total of nine plots. Plots measured 9.6 × 9.6 m and contained 12 rows with 12 plants in each row with offset every second row, resulting in a hexagonal planting pattern with equal distances of 0.8 m between individuals. This spacing corresponds to approximately 15,600 plants ha^−1^. Further details about the establishment of the sites, soil, and climatic conditions can be found in Hoeber et al. (2018). Soil temperature and precipitation for the experimental period at both sites are reported in Supplementary Figure 1.

### Soil Sampling

For molecular analysis, soil cores (3 × 10 cm) were sampled in April 2014, 2015, and 2017, and in April, August, and November 2016, covering one short rotation coppice cycle from planting to harvesting (2014–2016), one sampling point after (2017), and seasonal variations for 1 year (2016). In each plot, a total of nine soil cores were collected and pooled, generating a total of 270 samples from Uppsala (45 samples per sampling occasion), and 54 samples from Rostock (nine samples per sampling occasion). Immediately after sampling, soil cores were either stored in a freezer and later freeze dried (Uppsala), or stored in the freezer and oven-dried at 45°C for 48 h (Rostock); after which, each dried sample was homogenized, using a pestle and mortar.

### Quantification of Fungal and Root Biomass

For soil samples in 2017, total fungal biomass was quantified from 0.3 g of a soil sample, using the fungal biomarker ergosterol. Ergosterol is the most common sterol of Ascomycota and Basidiomycota. Using established methods (Nylund and Wallander, 1992), esterified ergosterol was extracted with 10% KOH in MeOH, filtered through a 0.45-μm Teflon filter, and 50 μl of each sample was analyzed, using high performance liquid chromatography, with a C18 reverse-phase column (Nova-Pak; 3.9 × 150 mm; Waters, Milford, CT, USA), preceded by a C18 reverse-phase guard column (Nova-Pak; 3.9 × 20 mm; Waters) as in Cheeke et al. (2017). The ergosterol peak was detected at 282 nm, using a UV detector. Fungal biomass was determined from ergosterol concentrations, using a conversion factor of a 3-μg ergosterol mg^−1^ dry sample (Salmanowicz and Nylund, 1988) and a correction factor (1/0.62) to compensate for unextracted ergosterol (Montgomery et al., 2000).

### Molecular Analysis

DNA was extracted from a subsample of approximately 400 μl by volume from each homogenized sample, using Macherey-Nagel NucleoSpin Soil kit (Düren, Germany), with the following modification; lysis buffer SL2 700 μl with 150 μl enhancer and quantified using a Thermo NanoDrop spectrophotometer (Wilmington, DE, USA). The internal transcribed spacer (ITS) region is the universal barcode for fungi, and ITS2 amplicons were produced, using the forward primer gITS7 (GTGARTCATCGARTCTTTG; (Ihrmark et al., 2012) and the two mixed reverse primers ITS4 (75%; 5′-TCCTCCGCTTATTGATATGC-3′; (White et al., 1990) and ITS4arch (25%; 5′-CACACGCTGTCCTCGCCTTATTGATATGC-3′), elongated with unique identification tags (Clemmensen et al., 2016). PCR was performed in 50 ml reactions with 25 μl DNA template (diluted to 0.5 ng/μl), 0.2 mM of each nucleotide, 0.75 mM MgCl_2_, forward primer at 0.5 μM, reverse primer at 0.3 μM, and 0.5 U polymerase (DreamTaq, Thermo Scientific, MA, USA) in PCR buffer on a 2720 Thermal Cycler (Life Technologies, California, USA). PCR conditions were 5 min at 94°C, 25–35 cycles of (30 s at 95°C, 30 s at 56°C, 30 s at 72°C) and 7 min at 72°C, and cycle numbers were adapted for each sample to give similar band strength to avoid oversaturation of the PCR pool. Two pooled PCR replicates from each sample were purified, using the AMPure kit (Beckman Coulter, California, USA) according to the instructions of the manufacturer, and quantified, using a Qubit® 2.0 fluorometer (Invitrogen, Carlsbad, California, USA). The products were mixed in equal amounts into four pools and cleaned, using the E.Z.N.A.® Cycle Pure Kit (Omega bio-tek, Norcross, Georgia, USA). Adaptor ligation and sequencing on PacBio Sequel instrument (Pacific Biosciences, Menlo park, California, USA) were performed by NGI-Uppsala/SciLifeLab (National Genomics Infrastructure, Uppsala, Sweden), using six Sequel SMRT cells (v2; pool 1) and two Sequel SMRT cells (v3; pools 2–4).

### Sequence Analyses

Raw sequence reads were analyzed, using the bioinformatics pipeline SCATA (https://scata.mykopat.slu.se) (Ihrmark et al., 2012). Sequences were quality filtered and screened for primers and identification tags as described in Kyaschenko et al. (2017) with some adjustments. After removal of sequences with mean quality of 20 bases and lower or containing bases with a quality of 3, sequences (complementary reversed, if needed) were searched for primers and identification tags. Only sequences containing matching tags at both ends were retained. All sequences were clustered into study-level species hypotheses (SHs; Koljalg et al., 2013), using a 1.2% threshold distance for sequences to enter an SH. A reference database from UNITE (UNITE community general FASTA release, version 7.2, release date, 10.10.2017) was included in the clustering. Sequences are available in the NCBI Sequence Read Archive (www.ncbi.nlm.nih.gov/sra) under the accession number PRJNA703824. To remove non-fungal sequences, the representative sequences from each SH were compared against GenBank nucleotide database using BLASTn, after which we used MEGAN (Huson et al., 2011) for the BLAST results and fasta file to assign the lowest common ancestor and to identify sequences that were not fungal (Balint et al., 2014). All singletons and the small clusters with five or less counts were removed. The representative sequences from each study-level SH were then compared to all global SHs, using the massBLASTer through the PlutoF platform in UNITE (Abarenkov et al., 2010) and assigned to appropriate a taxonomic level (at least 97% similarity was required for species-level identification, 90% for genus, 85% for family, 80% for order, 75% for class, and 70% for division/phylum), in order of decreasing global relative abundance until 80% of the sequences were covered. To ensure that the difference in sequencing chemistry (pool 1 vs. other pools) did not affect the outcome of community compositional analyses due to different sequencing depth, both the original study-level SHs table for all clusters and counts data, and the identified study-level SHs, were rarefied (100 random subsampling) to 320 and 168 sequences per sample, respectively. Samples with less sequences were removed (eight samples for all study-level SHs, 10 for the identified). Additionally, we applied centered log-ratio (clr) transformations (Gloor et al., 2017; Quinn et al., 2018) to the original study-level SHs table to obtain values that were scale-invariant and to account for differences in count numbers. Study-level SHs were further assigned to ecological functions, using FUNGuild software (Nguyen et al., 2016). In the functional group bar charts, all available functional groups (guilds) from FUNGuild were included with some modifications: SHs classified as fungal parasite-saprotroph and fungal parasite-undefined saprotroph were called fungal parasite/saprotroph; fungal parasite-protistan parasite was called putative fungal parasite; plant pathogen-plant saprotroph/undefined saprotroph/wood saprotroph combinations were called plant pathogen/saprotroph; endophyte-litter saprotroph/soil saprotroph/undefined saprotroph/wood saprotroph were called putative endophyte/saprotroph; combinations of different saprotrophic classification were called saprotrophs, and any other SHs with a functional classification with several possibilities were assigned as unknown. See Supplementary Table 1 for functional groups and exploration type assignments (Agerer, 2001, 2006; Tedersoo et al., 2006; Lilleskov et al., 2011; Katanic et al., 2014), and Supplementary Table 2 for relative abundances of the 446 most common identified SHs (rarefied and not rarefied data). In multivariate plots, showing the most common SHs (see below), functional groups were simplified, using ECM, saprotroph, plant pathogen, animal pathogen, and unknown.

### Statistical Analyses

Ordination analyses were performed using CANOCO version 5.02 (Microcomputer Power, Ithaca, NY, USA). Variation in soil fungal community composition (total fungal community; 446 SHs, and ECM fungal community; 18 SHs, see Supplementary Tables 2, 3) was visualized, using principal components analysis (PCA) and detrended correspondence analysis (DCA). Separate multivariate analyses were done for Rostock and Uppsala, as well as Rostock and Uppsala together, based on the individual samples, using both the rarefied (reported in the results) and the original data (supplemental data in Supplementary Material). In order to further control for differences in count numbers between samples, we also did multivariate analyses using clr-transformed data (not reported). The PCAs and DCAs reported were based on identified communities resolved at the SHs level, but additional analyses (not reported) were done with total fungal communities, including all study-level SHs to confirm that they produced the same pattern as the subset of identified SHs. Correlation between plant, fungal, and soil parameters with site, block, genotypes, and diversity levels, and their interaction with the identified soil fungal community (rarefied data) were tested, using redundancy analysis (RDA). We used combinations of previously published data from the same field trials on plant biomass (shoot: Hoeber et al., 2018, leaf biomass: Weih et al., 2021), leaf chemistry after one growing season (N, C, phosphorous (P), and lignin (g/kg), collected in autumn, 2014; Hoeber et al., 2020), and litter decomposition (chemistry of decomposed litter after 1.5- and 2-year soil incubation: N, C (g/kg) and ash fraction (weight of ash/weight of decomposed litter); Hoeber et al., 2020) together with soil properties of pre-planted fields (pH, N-, C-, S-content (%) and C:N ratio; Supplementary Table 4) and fungal biomass from 2017 (Table 1). These potential drivers were tested against the fungal community data for the corresponding sampling time. Simple term effects are reported after Bonferroni corrections.

**Table 1:** Fungal biomass (mean ± SD) per genotype treatment in 2017 for Rostock and Uppsala.

**Site**	**Diversity level**	**Genotype**	**Fungal biomass**
		**mixtures^***a***^**	**(mg/g soil)^***b***^**
Rostock	Monoculture	C	0.77 ± 0.07
	Monoculture	D	0.81 ± 0.02
	2-mixture	CD	0.81 ± 0.02
Uppsala	Monoculture	A	1.10 ± 0.15
	Monoculture	B	1.07 ± 0.26
	Monoculture	C	0.92 ± 0.14
	Monoculture	D	0.97 ± 0.04
	2-mixture	AB	0.94 ± 0.10
	2-mixture	AC	1.03 ± 0.13
	2-mixture	AD	1.00 ± 0.16
	2-mixture	BC	1.02 ± 0.08
	2-mixture	BD	1.10 ± 0.24
	2-mixture	CD	0.96 ± 0.08
	3-mixture	ABC	1.10 ± 0.06
	3-mixture	ABD	1.01 ± 0.04
	3-mixture	ACD	0.90 ± 0.07
	3-mixture	BCD	1.00 ± 0.12
	4-mixture	ABCD	1.08 ± 0.06

*Soil sampling for fungal biomass was done in April 2017, at the end of the first short rotation coppice cycle.*

a *Genotypes correspond to A = “Björn”, B = “Jorr”, C = “Loden”, and D = “Tora”.*

b *Fungal biomass was estimated from esterified ergosterol concentrations*.

Diversity measurements (species richness; i.e., total counts of SHs, Shannon-Wiener index and Simpson's index of diversity 1-D) based on the rarefied data for all study-level SHs are reported both as averages (± SD) per treatment (Supplementary Table 5; site × year × harvest time × diversity level × genotype × block) and for all individual samples (Supplementary Table 6). Species data were arcsine transformed before all multivariate analyses, with the exception of the identified SHs from Uppsala (original data, not rarefied), where species data were log transformed. Rare SHs were down-weighted in the DCA of the total fungal community analysis. We also used the multi-response permutation procedure (MRPP), a non-parametric procedure in PC-ORD version 5.33 software (McCune, 2006) for testing the hypothesis of no difference between two or more *a priori* assigned groups (McCune and Grace, 2002). This was done to test for the effects of genotype combination, presence of one genotype (“Björn,” “Jorr,” “Loden” or “Tora”), diversity level (monoculture, 2-, 3-, and 4-mixture), year (2014, 2015, 2016, and 2017), harvest time (April, August, and November), year × harvest time, and block on compositional data for Uppsala and Rostock (total and identified community, rarefied data) separately. The effects of site, diversity level, year, genotype combination, block, and harvest time on fungal biomass and diversity were tested, using R (version 3.6.1, R Core Team, 2019). Multiple comparison analyses were performed, using two-way ANOVA, followed by Tukey's HSD test. To summarize general temporal trends in community composition, functional groups were aggregated into two broad root-associated and saprotroph categories by summing up abundances as follows: root-associated included arbuscular mycorrhizal (two clusters), ectomycorrhizal, and endophyte (one cluster) groups; saprotrophs included endophyte-undefined saprotroph-wood saprotroph, all plant saprotroph, undefined saprotroph, and wood saprotroph groups. The abundances in these two groups were then normalized by the total abundance to calculate proportions of root-associated fungi and saprotrophs. Temporal trends were assessed for the root-associated and saprotroph proportions and the root-associated: saprotroph ratio, using a linear mixed effect model (*fitlme* function in Matlab R2018b, The Mathworks) initially including year, month, and number of *Salix* genotypes as fixed effects and the site as random effect. Since the number of genotypes had no predictive power, it was removed from the regression.

## Results

### Fungal Biomass

Fungal biomass for the genotypes “Loden” and “Tora” and their mixture present at both sites were significantly higher in Uppsala (1. ±0.02 mg g^−1^ soil) compared to Rostock (0.8 ±0.02 mg g^−1^ soil) (Table 1 and Supplementary Table 7). There were no differences in fungal biomass between different plant genotypes or diversity levels for either site.

### Sequencing Output

**Figure 1:**
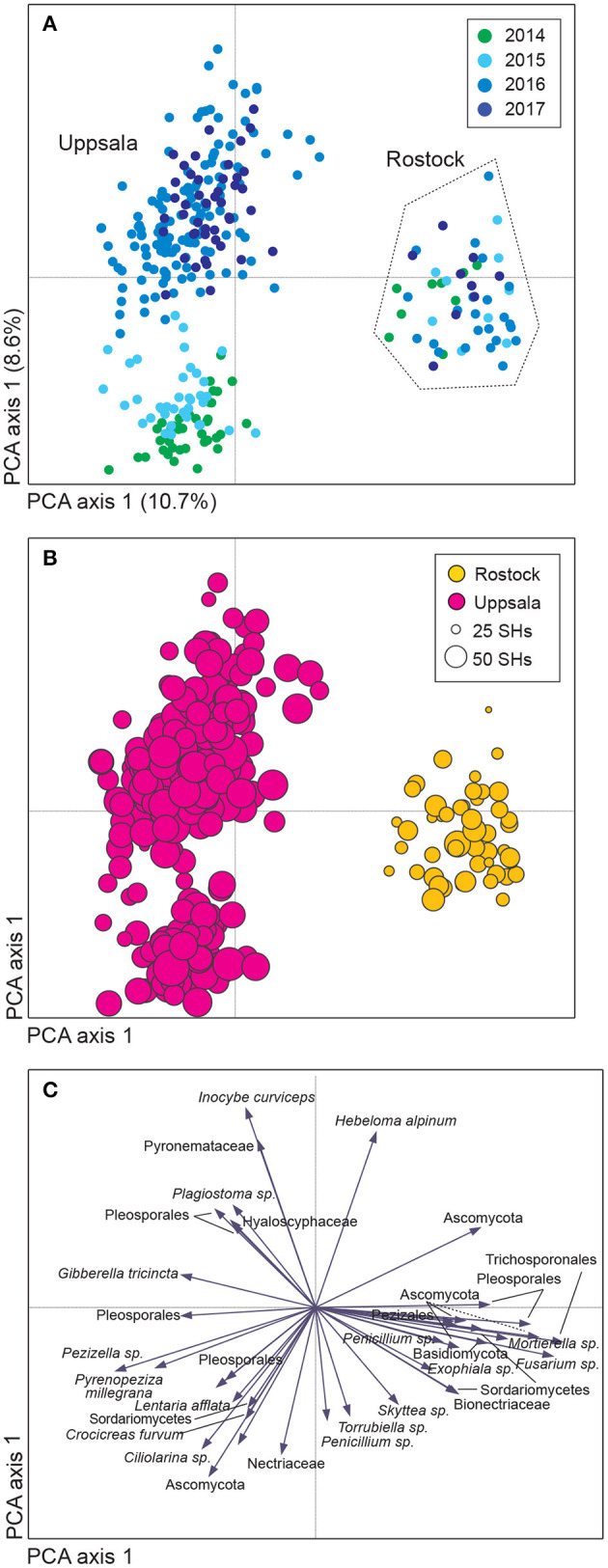
Variation in soil fungal community composition in *Salix* genotype trials with the four genotypes “Björn,” “Jorr,” “Loden,” and “Tora” planted in monoculture and various genotype mixtures (2-, 3-, and 4-mixtures) at two different sites (Rostock, Germany, and Uppsala, Sweden). Community composition is visualized by **(A,B)** a sample plot and **(C)** a species plot of a principle component analysis (PCA) based on PacBio sequencing of amplified ITS2 markers. The PCA was based on 446 identified fungal SHs, rarefied data. Circles are color coded according to: **(A)** year and **(B)** site with circle area indicating number of species hypotheses (SHs) in each sample. In **(C)**, only the 40 most abundant SHs are shown. Axes 1 and 2 explained 10.7 and 8.6%, respectively, of the total inertia of 1.5.

Sequencing generated a total of 2,294,094 reads, of which 1,340,101 reads passed quality control (QC). After removal of non-fungal sequences (427,706 reads, 32% of reads passed QC) and singletons (23,483 reads), the remaining 888,912 reads clustered into 4,839 study-level SHs. Clusters with five or fewer reads (2,400 clusters and 6,568 reads) were removed, resulting in a total of 2,421 study-level SHs; out of which, 446 SHs (80% of the fungal sequences) were subjected to taxonomic and functional identification (Supplementary Tables 1, 2). Each sample had an average of 287 reads (3,023 maximum, 200 minimum). After rarefying the dataset, 1,943 and 446 study-level SHs remained for all and identified clusters, respectively. Although the number of sequences per sample varied greatly (Supplementary Figure 2), it was clear from the multivariate analyses that the rarefied and the original dataset (Figure 1, Supplementary Figures 3, 4), as well as the clr-transformed dataset (not reported), showed very similar community compositional patterns.

### Fungal Functional Groups and Diversity

**Figure 2:**
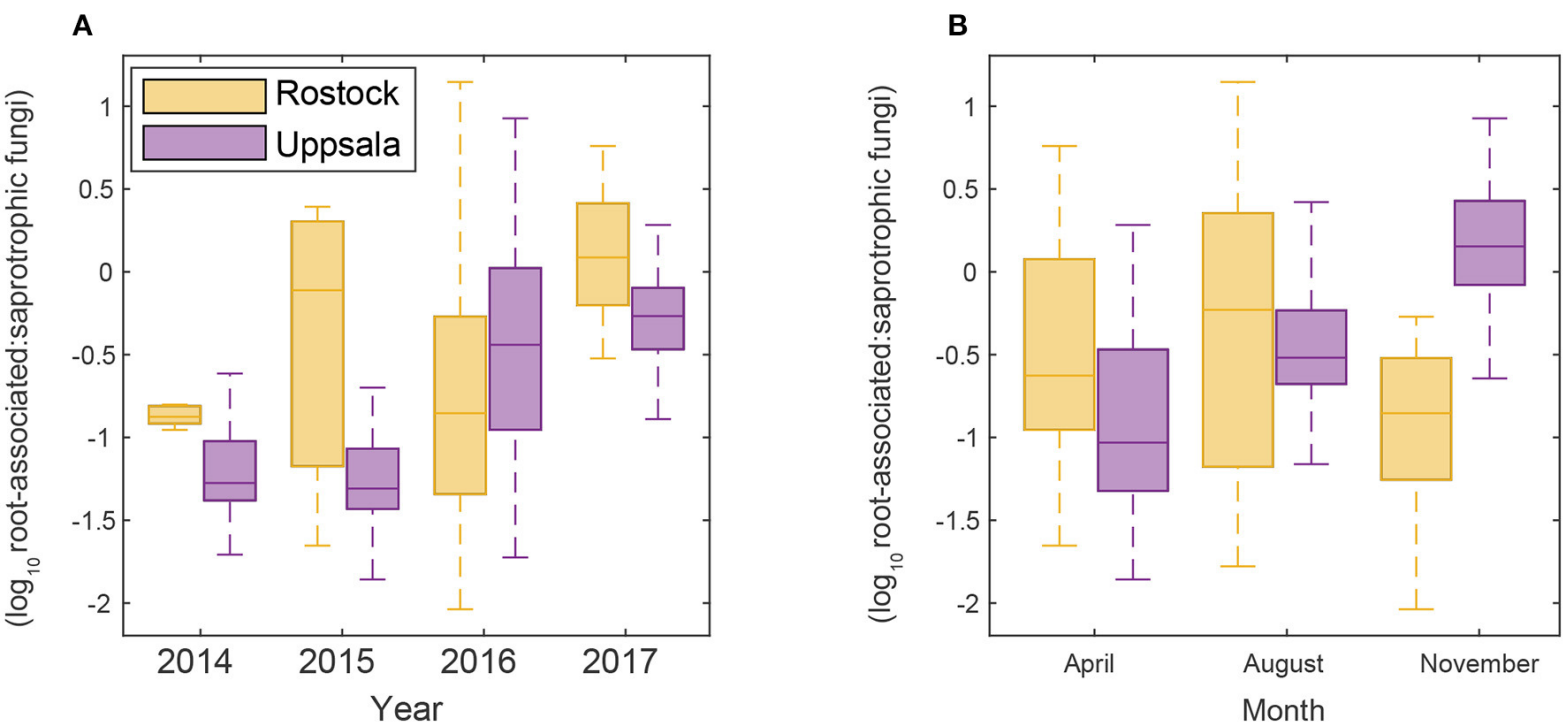
Changes in the proportion of a root-associated over saprotrophic sequence counts **(A)** throughout time over the study period and **(B)** seasonally in Rostock and Uppsala.

**Figure 3:**
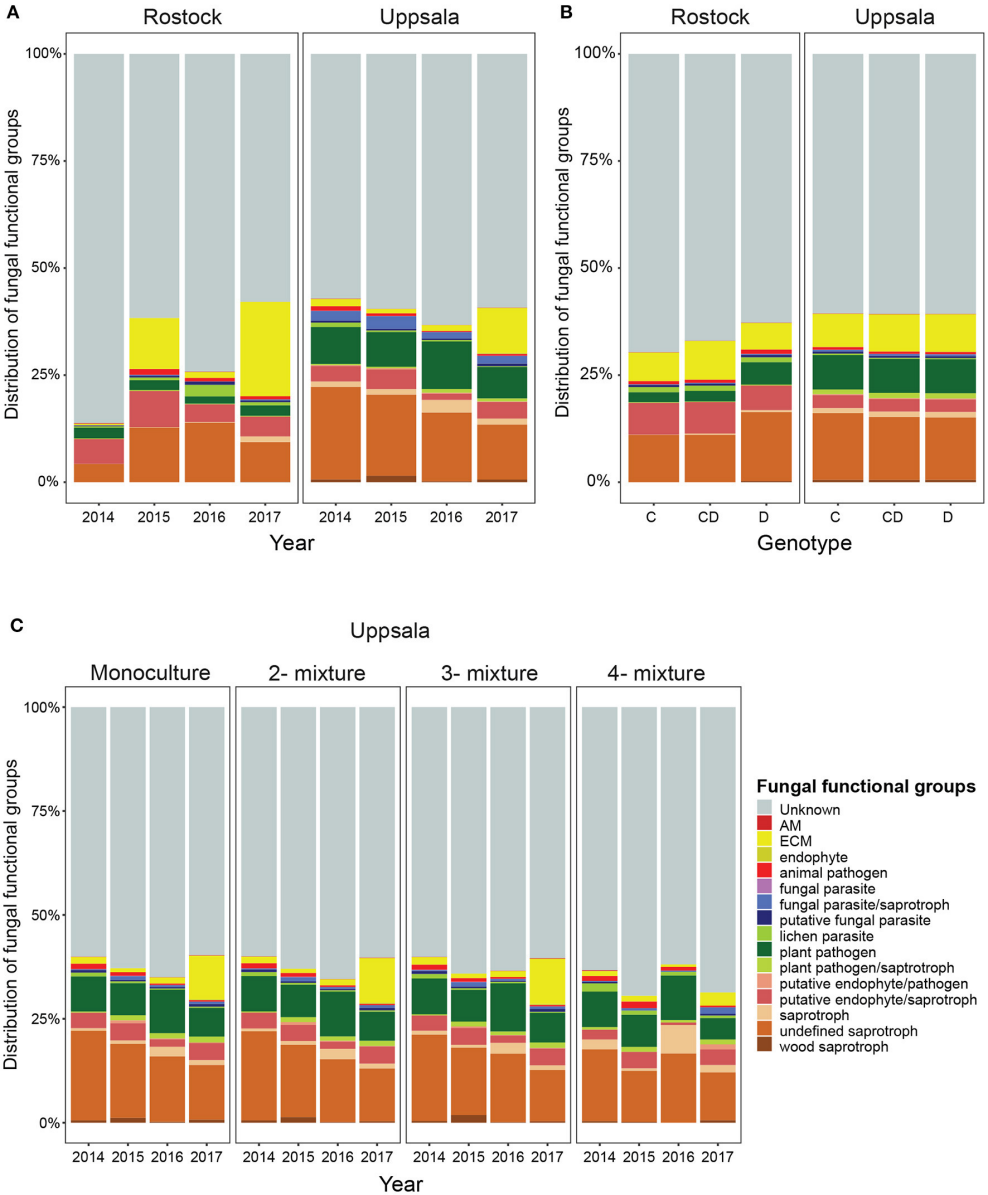
Distribution of fungal functional groups in soil from *Salix* genotype trials in Rostock, Germany, and Uppsala, Sweden: **(A)** over time (preplanting in 2014 to 2017) **(B)** in monocultures of “Loden” or “Tora” and in 2-mixtures of “Loden” and “Tora”, and **(C)** for Uppsala only, in monoculture, 2-, 3-, and 4-mixtures, as estimated by PacBio sequencing of amplified ITS2 markers. Abundances are given as percent of the identified amplicon sequences, based on rarefied data (accounting for 80% of total sequences). There was a large increase in the proportion of ECM fungi three years after planting at both sites, especially in monoculture, 2- and 3-mixtures in Uppsala but with a smaller increase in 4-mixtures.

Uppsala and Rostock were colonized by distinct soil fungal communities (Figure 1 and Supplementary Figure 3; Supplementary Table 2). For the functional groups, the proportion of ECM fungi increased significantly over time (from preplanting until the first short rotation coppice cycle ended) irrespective of the plant diversity level (monoculture, 2-, 3-, or 4-mixture), and was followed by a significant decrease in the proportion of saprotrophic fungi (Figure 2, Table 2). ECM fungi increased about 6-fold in Uppsala and 70-fold in Rostock 3 years after planting at both sites (Figure 3A), but this large increase in Uppsala was only observed in monocultures 2- and 3-mixtures, while the 4-mixture showed a weaker response (Figure 3C). The 2-mixture of genotypes “Loden” and “Tora” in Rostock showed an increase of ECM fungi by ca 55% compared to the monocultures (Figure 3B). Fungal diversity (Supplementary Tables 5, 6) did not differ depending on the plant diversity level or genotype mixture (Supplementary Table 8). Species richness was significantly higher in Uppsala compared to Rostock (Figure 1B, Supplementary Tables 5, 6, 8), with an average of 94 ± 1 and 69 ± 2 SHs, respectively, taken over all treatments and times. Although fungal species richness was variable between years within individual genotype treatments, there was an increase in species diversity over time for some of the genotypes for both sites (Figure 1, Supplementary Tables 5, 8).

**Table 2:** Summary of results from linear mixed effect models to predict root-associated and saprotrophic contributions to the total sequence counts, and the ratio of root-associated over saprotrophic counts.

	**Significance of “year” effect**	**Significance of “month” effect**	**Ordinary coefficient of determination (R^**2**^)**
Proportion of root-associated counts	>0**	>0**	0.35
Proportion of saprotrophic counts	<0**	<0*	0.07
Ratio of root-associated over saprotrophic counts (log transformed)	>0**	>0**	0.43

In all cases, linear models include year and month as fixed effects, and site as random effect [Y ~ 1 + Year + Month + (1 | Site)]; only the signs of the effects and their significance levels (*p < 0.05, **p < 0.001) are reported.

### Potential Drivers of Community Responses

**Figure 4:**
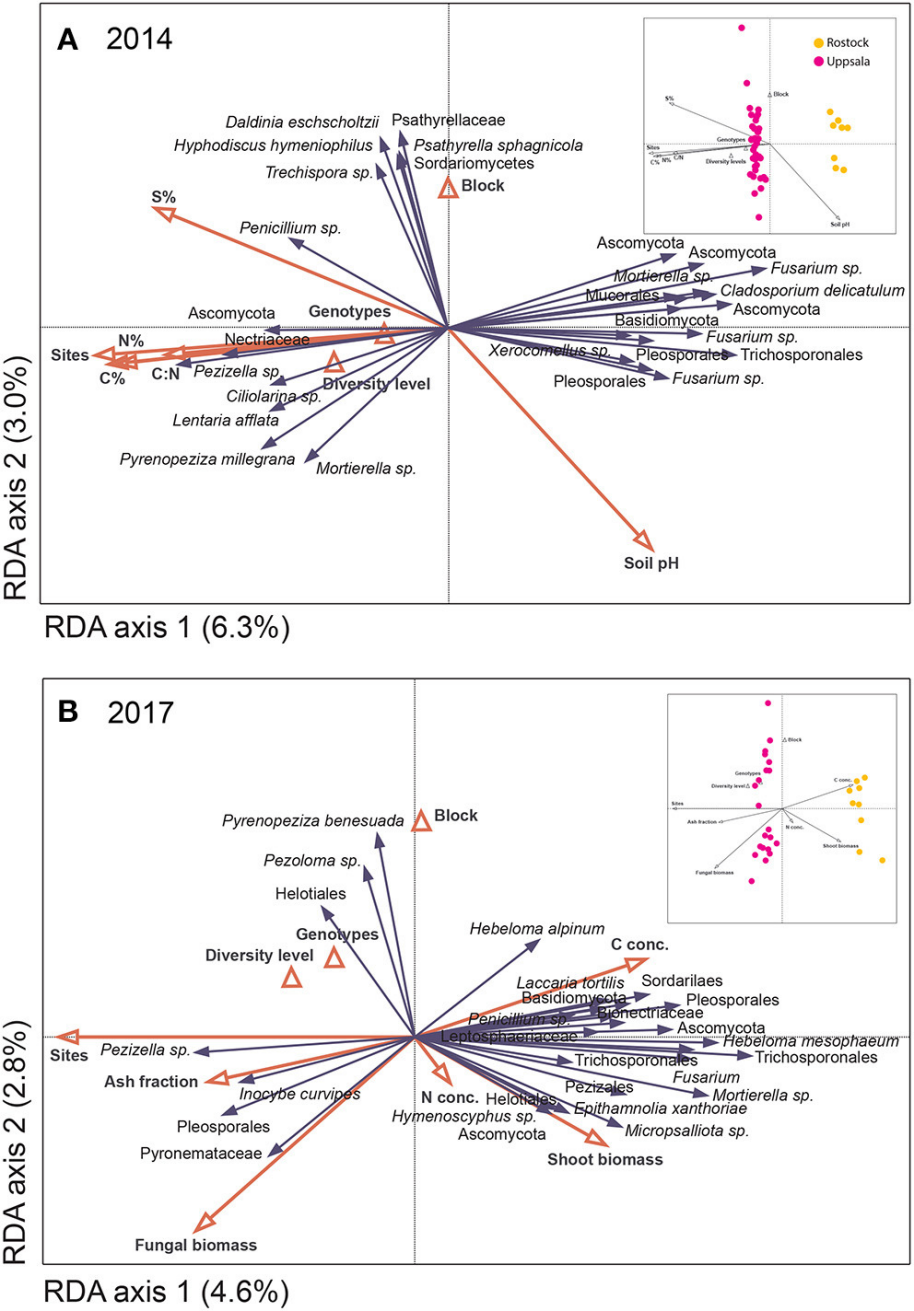
Distribution of fungal species hypotheses (SHs) in *Salix* genotype trials in Uppsala and Rostock in **(A)** 2014 and **(B)** 2017, as visualized by species plots of redundancy analyses (RDA) based on PacBio sequencing of amplified ITS2 markers. The RDAs included 442 (Uppsala) and 217 (Rostock) identified SHs, but only the 30 most abundant fungal species are shown. The triangles and red vectors represent constraining environmental variables. In 2014, axes 1 and 2 explained 6.3 and 3.0%, respectively, of the total inertia of 13,296. N%, C%, S%, and C:N ratio correspond to soil properties. In 2017, axes 1 and 2 explained 4.6 and 2.8%, respectively, of the total inertia of 20,250. N- and C-concentrations and ash fraction correspond to litter chemistry from a litter decomposition experiment. Explanatory variables accounted for 22.9% (2014) and 19.9% (2017). Inserts show sample plots of the RDAs.

The overall community composition was significantly related to site, soil properties of pre-planted fields, fungal biomass in soil, plant shoot biomass, some of the leaf chemistry variables, and some of the litter decomposition variables (see Figure 4 for analyses of 2014 and 2017 data, 2015 and 2016 not shown). The potential drivers together explained 20–40% of the variation in fungal community composition. Site was always the explanatory variable, accounting for most of the variation in fungal community composition (*p* ≤ 0.026 up to 13.5%; Figure 4). Soil properties of pre-planted fields significantly explained part of the variation (*p* = 0.018, ca 5% of the variation for each variable) for the fungal community in 2014 (Figure 4A) and the following years, although explaining less of the variation over time (not shown). Leaf N and P concentrations after one growing season were the only leaf chemical characteristics that weakly explained community composition in 2015 (*p* = 0.032 and 0.016, respectively; not shown). Fungal biomass in soil, plant shoot biomass, and some of the litter decomposition variables (C-concentration and ash fraction) in 2017 each explained about 3% of the variation (*p* ≤ 0.036; Figure 4B). Furthermore, in Rostock, there were significant effects of the diversity level (*p* <0.036, A = 0.0086), genotype (*p* <0.018, A = 0.014), and presence of genotype “Loden” (*p* < 0.013, A = 0.012) on community composition of *a priori* assigned groups.

### Year-to-Year and Seasonal Variation in Community Composition and Diversity

**Figure 5:**
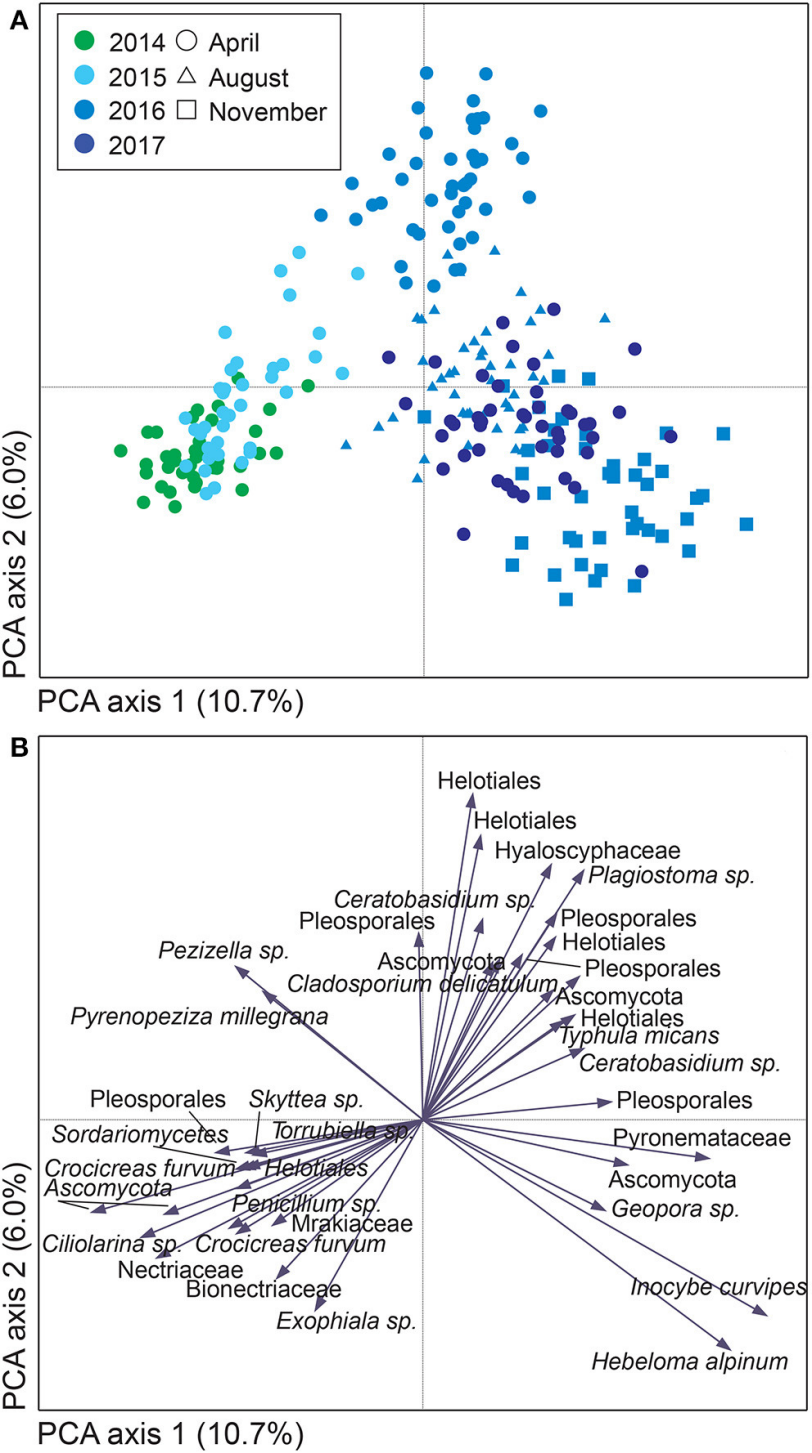
Variation in soil fungal community composition in *Salix* genotype trial with four genotypes “Björn,” “Jorr,” “Loden” and “Tora” planted in monoculture and various genotype mixtures (2-, 3-, and 4-mixtures) in Uppsala, Sweden. Community composition is visualized by **(A)** a sample plot and **(B)** a species plot of a principle component analysis (PCA) based on PacBio sequencing of amplified ITS2 markers. The PCA was based on 442 identified fungal SHs, rarefied data. Symbols are color coded according to: **(A)** year, with symbol shape indicating sampling month. In **(B)**, only the 40 most abundant SHs are shown. Axes 1 and 2 explained 10.7 and 6.0%, respectively, of a total inertia of 1.2.

**Figure 6:**
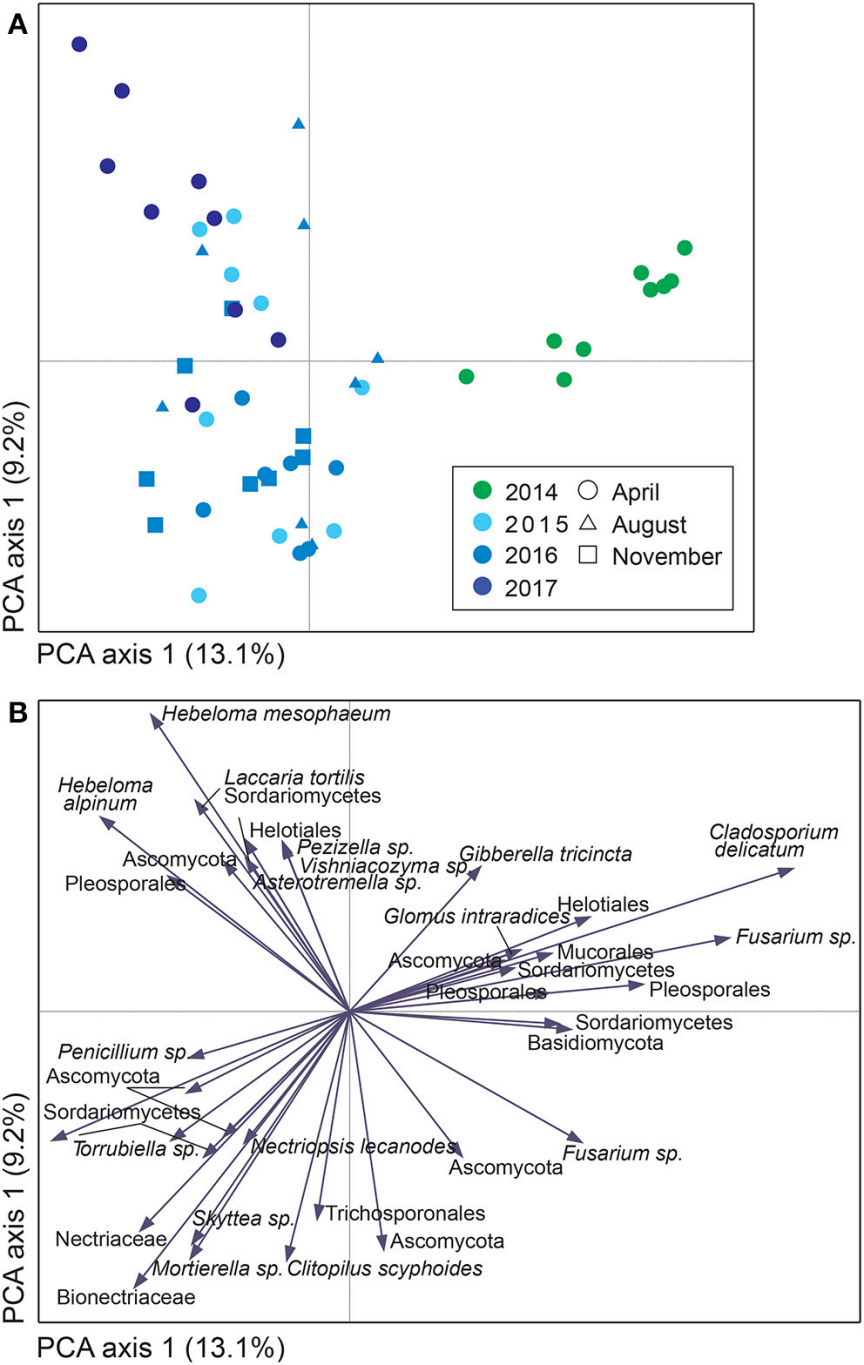
Variation in soil fungal community composition in *Salix* genotype trial with two genotypes (“Loden” and “Tora”) planted in monocultures and 2-mixture in Rostock, Germany, visualized by **(A)** a sample plot and **(B)** a sample plot of a principle component analysis (PCA) and based on PacBio sequencing of amplified ITS2 markers. The PCA was based on 217 identified fungal SHs, rarefied data. Symbols in **(A)** are color coded according to year, with symbol shape, indicating sampling month and area, indicating total number of SHs in each sample. Axes 1 and 2 explained 13.1 and 9.2%, respectively, of a total inertia of 0.2.

For both sites, the fungal community changed over time; in Uppsala (Figure 5 and Supplementary Figure 5), 1 year after planting the composition was similar to pre-planting and dominated by SHs in Ascomycota (Helotiales, *Torrubiella* sp., *Ciliolarinia* sp., *Crocicreas furvum*, and *Penicillium* sp.); after which, other ascomycetes became more common alongside with *Mortierella* sp. and basidiomycetes (*Inocybe curviceps* and *Hebeloma alpinum*). In Rostock, the community in pre-planted soil was separated from planted and developed over time (Figure 6 and Supplementary Figure 6) but with a more similar community structure compared to Uppsala. Pre-planting, SHs beloning predominantly to Ascomycota (Helotiales, *Cladoaporium* sp., and *Fusarium* sp.; Figure 6B) were most common. After planting, ECM fungi (*Hebeloma alpinum, Hebeloma mesophaeum*, and *Laccariatortilis*) and other basidiomycetes (*Lentaria afflata* and *Clitopilus hobsonii*) became more common (Figure 6B). The within-season variation was of a similar magnitude as the year-to-year variation in Uppsala (Figure 5A) and was also apparent in Rostock, with August showing the largest spread in composition (Figure 6A). Species richness (Supplementary Table 8) and the proportion of ECM fungi (Supplementary Figure 7) were highest in November in Uppsala. In Rostock, the proportion of ECM fungi varied strongly between years, and after the third growing season ECM fungi contributed almost 25% to the total fungal community (Figure 3A). There were significant effects of year (MRPP Uppsala: *p* < 10^−9^, A = 0.11; Rostock: *p* < 10^−9^, A = 0.10), and block (Uppsala: *p* < 10^−9^, A = 0.0074) on community composition of *a priori* assigned groups. Block 3 diverged somewhat in community composition from other blocks (Supplementary Figure 8). For the ECM community in Uppsala, development over time was similar to the fungal community, but less pronounced, and ECM species richness also increased over time (Supplementary Figure 9). Specifically, species richness was significantly higher in 2017 compared with both 2015 and 2016 (Supplementary Table 8).

## Discussion

In this study, we investigated the interactive responses of soil fungal community composition to plant genotypic diversity and environmental drivers in a *Salix* biomass system. Although there was no overall effect of genotype, we found some site-dependent relationships between fungal community composition and plant genotypic diversity, and site accounted for the largest part of the variation in fungal community composition. In Rostock, where the fungal community structure was more homogenous compared to in Uppsala, there were significant effects of genotype, diversity level, and the presence of one genotype (“Loden”) on fungal community composition. The proportion of ECM fungi increased over time, and, in Uppsala, the 4-mixture showed a weaker response than other genotype combinations. Overall, fungal communities were thus site-dependent in relation to plant community diversity. However, the ECOLINK-*Salix* trials would need to be studied longer and also include more genotypes, since potential genotype effects on plant, microbial, and soil parameters may become apparent in later short rotation coppice cycles.

### Few Effects of Plant Genotype Diversity on Soil Fungal Diversity

For answering our first research question about how fungal diversity varies as a function of plant diversity, we found some support for plant genotype effects in the site-specific analyses where fungal diversity increased in the 2-mixture of “Loden” and “Tora” compared to the monocultures in Rostock (MRPP analysis), suggesting that the plant genotype diversity effect is site or context dependent (Tedersoo et al., 2016; Bonito et al., 2019). A common garden experiment, using a *Populus* model system, found a host genotype to explain as much as 70% of the variation in microbial community composition based on PLFAs (Schweitzer et al., 2008); however, this large difference in plant genotype effects on microbial communities was most likely explained by minimization of site bias and the use of the method to characterize the microbial community. In the present study, both sites were characterized by distinct soil fungal communities with a high number of SHs, comparable to what previous community studies have reported in terms of composition, dominating taxa and mean number of taxa (Perez-Izquierdo et al., 2017; *Pinus pinaster*), although Rostock had significantly lower species richness compared with Uppsala. Since soil microbial diversity in general is linked to the clay content (Xue et al., 2018), which is much higher in Uppsala compared with Rostock (Hoeber et al., 2020), we would still expect higher fungal diversity in Uppsala (Essene et al., 2017). A low ECM and endophyte diversity in roots in all genotype treatments in the Rostock trial were also shown previously (Baum et al., 2018), but the mycorrhizal root tips did not show a strong increase in ECM colonization in the 2-mixture compared to monocultures as the increase in the proportion ECM fungi in soil in the Rostock 2-mixture in the present study. In Uppsala, site conditions were more heterogeneous (block effects). The large increase in the proportion of ECM fungi over time was confined to monocultures, 2- and 3-mixtures, and much lower in the 4-mixtures, suggesting that there could be an “optimal” genotype mixture or a plant diversity level for sustaining ECM fungal communities. Similarly, the 4-mixture tended to exhibit slower litter decomposition compared to monocultures, 2- and 3-mixtures, in Uppsala and Freiburg (the third ECOLINK site, not included in the present study; Hoeber et al., 2020). Tree productivity results for Freiburg also showed that shoot biomass production was more variable and lower in the 4-mixture compared with the less diverse stands (Hoeber et al., 2018). These findings also corroborate previous studies, showing that higher tree or genotype diversity can have diverse impacts on fungal diversity and composition (e.g., Baum et al., 2018; Bonito et al., 2019).

### Plant Genotype Identity and Other Drivers of Fungal Communities

Plant genotype identity was not a strong driver of fungal community composition in line with previous studies (Erlandson et al., 2016, 2018; Arraiano-Castilho et al., 2020) – the only evidence was the significant effect of “Loden” on fungal community composition in Rostock. The different genotypes in the ECOLINK-Salix trial partly belong to different *Salix* species and have contrasting phenology, functional traits, and ecophysiology (Weih and Nordh, 2002; Weih et al., 2021). Although increasing genotype diversity so far did not significantly affect aboveground plant productivity in young stands, admixing of two genotypes (“Jorr” and “Loden”) was predicted to enhance the total shoot biomass production in a mixed stand, while two other genotypes (“Tora” and “Björn”) were more likely to reduce the total shoot biomass production in mixtures (Hoeber et al., 2018). Hence, we expected to find similar effects on soil fungal communities, because of the expected links between productivity and belowground communities. The effect on fungal communities of having “Loden” present (in Rostock) corroborates the potential beneficial effect of “Loden” on biomass production in mixed stands, since a larger tree biomass production in stands in which “Loden” (as opposed to “Tora”) is admixed may possibly be linked to more belowground C, supporting soil fungal communities. This possible mechanistic link would, however, require verification by means of further experimental work.

Since the relative importance of interactions between stand characteristics, soil properties, and climatic conditions in relation to tree genotype diversity or identity is hardly known so far, we also tested the significance of plant genotypic diversity on the soil fungal community composition in relation to other drivers (soil properties, plant biomass, litter chemistry, litter decomposition, and fungal biomass). Although several of the plant derived properties and soil properties were significantly correlated with soil fungal communities, we found no significant overall effects of plant genotype mixtures. These results support the idea that the different drivers acted in concert dependent on site – but *not* always *via* plant genotype differences – to shift fungal community composition and diversity in the present study. This contrasts with the idea that genotypic variation in aboveground plant traits is important to soil microbial dynamics and nutrient availability (Schweitzer et al., 2004). ECM fungal communities have, for example, been linked to plant biomass (growth and size of host plants; Korkama et al., 2006; Velmala et al., 2013) and leaf chemistry (ECM community covary with senescent leaf chemistry; Lamit et al., 2016). One study also found no plant genotype effect, reporting on diverse and evenly distributed soil fungal communities in different *Populus* genotypes (Danielsen et al., 2012). Soil chemistry also can be a strong driver of fungal communities (Gallart et al., 2018b; Bonito et al., 2019), especially nutrient availability and soil pH.

### Year-to-Year Variation in Fungal Community Composition as Large as Seasonal Variation

We expected the variation in fungal community composition within the season to be larger than the year-to-year variation, since within-season variation in environmental conditions and plant inputs is often higher than year-to-year changes. Instead, the within-season variation was of a similar magnitude as the year-to-year variation, although community composition in Uppsala varied more than in Rostock, possibly due to a wider seasonal temperature range and greater spatial heterogeneity. Large within-season variation in fungal community composition has been frequently reported (Voriskova et al., 2014; Haas et al., 2018). For example, Baum and Hrynkiewicz (2006) found a seasonal shift in saprotrophic fungi and enzymatic activities associated with two *Salix* genotypes, and, although community composition shifted over the growing season, diversity remained larger for one of the genotypes. Variation in the root microbiome of *Populus* trees was explained by season and soil properties (Shakya et al., 2013). Therefore, interannual variation seems to have less importance compared with seasonal variation on timescales shorter than successional scales, e.g., for ECM fungi (Bahram et al., 2015, and references therein). However, over longer timescales, the fungal communities undergo significant successional changes. Indeed, we found an increase in both fungal diversity and proportion of ECM fungi over time. An increase in the proportion of ECM fungi, after establishing mycorrhizal *Salix* on arable land, would be expected (Dickie et al., 2013), and is analogous to forest regrowth after clear-cut (see, e.g., Kyaschenko et al., 2017). As plants grow, they supply the surrounding soil with C, influencing soil chemistry and biota (Leake and Read, 2017), and larger trees have the capacity to support more symbiotic fungi. Since tree biomass was larger in Rostock compared with Uppsala (mean shoot biomass production over the whole cycle after the first cutting, 8.7 Mg ha^−1^ and 5.7 Mg ha^−1^ in Rostock and Uppsala, respectively), this difference in tree biomass likely explains the higher proportion of ECM fungi found in Rostock.

### Methodological Considerations

The potential problem with differences in sequencing depths due to sequencing chemistry (amplicon pool 1: 276,111 sequences, 25% passing QC; amplicon pools 2–4: mean number of sequences 672,661, 63% passing QC; see Materials and Methods) was handled by rarefying data. AM fungi were only detected infrequently in our study, even though *Salix* was planted on a former agricultural field where they should be present and even promoted by the no-till management of *Salix*. Low AM fungal colonization of *Salix* has been described by several authors (e.g., Püttsepp et al., 2004; Hrynkiewicz et al., 2012). Furthermore, low detection of AM fungi may also be due to low abundance (in terms of DNA) relative to other fungal taxa, or because the analytical methods used to quantify fungi (ITS2 primers) were not optimal for this fungal group (e.g., Delavaux et al., 2020). Although these primers work well for amplifying the fungal ITS2 region for Ascomycota and Basidiomycota (Ihrmark et al., 2012), they are less frequently used in AM community studies (Glomeromycota) due to poor amplification (Tedersoo et al., 2018). Furthermore, sampling soil instead of mycorrhizal roots may be masking direct genotype effects on root-associated fungi. It should also be stressed that our results are correlative and that manipulative experiments would be required to consolidate our conclusions and confirm causal relationships.

## Conclusions

*Salix* spp. in short rotation coppice was here successfully used as model system to explain environmental changes in soil ecological properties, especially fungal diversity. Fungal communities were largely independent of plant community diversity but were affected mainly by site-specific conditions. This significant site specificity underlines the need of consideration of diverse sites to draw general conclusions on temporal variations and functioning of fungal communities. A significant increase in ECM colonization of soil under the pioneer tree *Salix* on agricultural soils was evident and points to a changed litter decomposition and soil C dynamics during *Salix* growth, where symbiotic nutrient exchange might be an important driver to consider.

## Data Availability Statement

The datasets presented in this study can be found in online repositories. The names of the repository/repositories and accession number(s) can be found in the article/Supplementary Material.

## Author Contributions

MW initiated the field experiment together with CB. SH and PF carried out field sampling. SH extracted soil DNA and prepared one sequencing pool. PF performed most sequence analyses and multivariate analyses. SH carried out other statistical analyses, using R. SM contributed to regression analysis. PF drafted the manuscript. SH, CB, MW, and SM contributed to data interpretations and article development, and approved the submitted version. All authors contributed to the article and approved the submitted version.

## Conflict of Interest

The authors declare that the research was conducted in the absence of any commercial or financial relationships that could be construed as a potential conflict of interest.

## Publisher's Note

All claims expressed in this article are solely those of the authors and do not necessarily represent those of their affiliated organizations, or those of the publisher, the editors and the reviewers. Any product that may be evaluated in this article, or claim that may be made by its manufacturer, is not guaranteed or endorsed by the publisher.

## References

[B1] Abarenkov K. Tedersoo L. Nilsson R. H. Vellak K. Saar I. Veldre V.. (2010). PlutoF-a web based workbench for ecological and taxonomic research, with an online implementation for fungal ITS sequences. Evol. Bioinform. 6, 189–196. 10.4137/EBO.S6271

[B2] Agerer R. (2001). Exploration types of ectomycorrhizae - A proposal to classify ectomycorrhizal mycelial systems according to their patterns of differentiation and putative ecological importance. Mycorrhiza 11, 107–114. 10.1007/s005720100108

[B3] Agerer R. (2006). Fungal relationships and structural identity of their ectomycorrhizae. Mycol. Prog. 5, 67–107. 10.1007/s11557-006-0505-x

[B4] Arraiano-Castilho R. Bidartondo M. I. Niskanen T. Zimmermann S. Frey B. Brunner I.. (2020). Plant-fungal interactions in hybrid zones: ectomycorrhizalcommunities of willows (Salix) in an alpine glacier forefield. Fungal Ecol. 45:100936. 10.1016/j.funeco.2020.100936

[B5] Baeten L. Bruelheide H. van der Plas F. Kambach S. Ratcliffe S. Jucker T.. (2019). Identifying the tree species compositions that maximize ecosystem functioning in European forests. J. App. Ecol. 56, 733–744. 10.1111/1365-2664.13308

[B6] Bahram M. Peay K. G. Tedersoo L. (2015). Local-scale biogeography and spatiotemporal variability in communities of mycorrhizal fungi. New Phytol. 205, 1454–1463. 10.1111/nph.13206 25767850

[B7] Balint M. Schmidt P. A. Sharma R. Thines M. Schmitt I. (2014). An illumina metabarcoding pipeline for fungi. Ecol. Evol. 4, 2642–2653. 10.1002/ece3.1107 25077016 PMC4113289

[B8] Baum C. Hrynkiewicz K. (2006). Clonal and seasonal shifts in communities of saprotrophic microfungi and soil enzyme activities in the mycorrhizosphere of Salix spp. J. Plant Nutr. Soil Sci. 169, 481–487. 10.1002/jpln.200521922

[B9] Baum C. Hrynkiewicz K. Szymanska S. Vitow N. Hoeber S. Fransson P. M. A.. (2018). Mixture of *Salix* genotypes promotes root colonization with dark septate endophytes and changes P cycling in the mycorrhizosphere. Front. Microbiol. 9:1012. 10.3389/fmicb.2018.01012 29867898 PMC5968087

[B10] Bonito G. Benucci G. M. N. Hameed K. Weighill D. Jones P. Chen K.-H.. (2019). Fungal-bacterial networks in the *Populus* rhizobiome are impacted by soil properties and host genotype. Front. Microbiol. 10:481. 10.3389/fmicb.2019.00481 30984119 PMC6450171

[B11] Bubner B. Fladung M. Lentzsch P. Muenzenberger B. Huettl R. F. (2013). Individual tree genotypes do not explain ectomycorrhizal biodiversity in soil cores of a pure stand of beech (*Fagus sylvatica* L.). Trees-Struct. Funct. 27, 1327–1338. 10.1007/s00468-013-0881-1

[B12] Cheeke T. E. Phillips R. P. Brzostek E. R. Rosling A. Bever J. D. Fransson P. (2017). Dominant mycorrhizal association of trees alters carbon and nutrient cycling by selecting for microbial groups with distinct enzyme function. New Phytol. 214, 432–442. 10.1111/nph.14343 27918073

[B13] Clemmensen K. E. Ihrmark K. Durling M. B. Lindahl B. D. (2016). Sample preparation for fungal community analysis by high-throughput sequencing of barcode amplicons in Microbial Environmental Genomics (MEG), ed S. U. F. Martin (New York, NY: Springer), 61–88. 10.1007/978-1-4939-3369-3_4 26791497

[B14] Cregger M. A. Veach A. M. Yang Z. K. Crouch M. J. Vilgalys R. Tuskan G. A.. (2018). The *Populus* holobiont: dissecting the effects of plant niches and genotype on the microbiome. Microbiome 6:31. 10.1186/s40168-018-0413-8 29433554 PMC5810025

[B15] Cunniff J. Purdy S. J. Barraclough T. J. P. Castle M. Maddison A. L. Jones L. E.. (2015). High yielding biomass genotypes of willow (*Salix* spp.) show differences in below ground biomass allocation. Biomass Bioenerg. 80, 114–127. 10.1016/j.biombioe.2015.04.020 26339128 PMC4547486

[B16] Danielsen L. Thurmer A. Meinicke P. Buee M. Morin E. Martin F.. (2012). Fungal soil communities in a young transgenic poplar plantation form a rich reservoir for fungal root communities. Ecol. Evol. 2, 1935–1948. 10.1002/ece3.305 22957194 PMC3433996

[B17] De Deyn G. B. Cornelissen J. H. C. Bardgett R. D. (2008). Plant functional traits and soil carbon sequestration in contrasting biomes. Ecol. Lett. 11, 516–531. 10.1111/j.1461-0248.2008.01164.x 18279352

[B18] Delavaux C. S. Sturmer S. L. Wagner M. R. Schütte U. Morton J. B. Bever J. D. (2020). Utility of large subunit for environmental sequencing of arbuscular mycorrhizal fungi: a new reference database and pipeline. New Phytol. 229, 3048–3052. 10.1111/nph.17080 33190292

[B19] Dickie I. A. Martinez-Garcia L. B. Koele N. Grelet G. A. Tylianakis J. M. Peltzer D. A.. (2013). Mycorrhizas and mycorrhizal fungal communities throughout ecosystem development. Plant Soil. 367, 11–39. 10.1007/s11104-013-1609-0

[B20] Elferjani R. DesRochers A. Tremblay F. (2014). Effects of mixing clones on hybrid poplar productivity, photosynthesis and root development in northeastern Canadian plantations. For. Ecol. Manag. 327, 157–166. 10.1016/j.foreco.2014.05.013

[B21] Erlandson S. Wei X. Savage J. Cavender-Bares J. Peay K. (2018). Soil abiotic variables are more important than Salicaceae phylogeny or habitat specialization in determining soil microbial community structure. Mol. Ecol. 27, 2007–2024. 10.1111/mec.14576 29603835

[B22] Erlandson S. R. Savage J. A. Cavender-Bares J. M. Peay K. G. (2016). Soil moisture and chemistry influence diversity ofectomycorrhizal fungal communities associating with willow along an hydrologic gradient. FEMS Microbiol. Ecol. 92:fiv148. 10.1093/femsec/fiv148 26622067

[B23] Essene A. L. Shek K. L. Lewis J. D. Peay K. G. McGuire K. L. (2017). Soil type has a stronger role than dipterocarp host species in shaping the ectomycorrhizal fungal community in a Bornean lowland tropical rain forest. Front. Plant Sci. 8:1828. 10.3389/fpls.2017.01828 29163567 PMC5663695

[B24] Gallart M. Adair K. L. Love J. Meason D. F. Clinton P. W. Xue J.. (2018a). Genotypic variation in *Pinus radiata* responses to nitrogen source are related to changes in the root microbiome. FEMS Microbiol. Ecol. 94, 1–15. 10.1093/femsec/fiy071 29688427

[B25] Gallart M. Adair K. L. Love J. Meason D. F. Clinton P. W. Xue J.. (2018b). Host genotype and nitrogen form shape the root microbiome of *Pinus radiata*. Microbial Ecol. 75, 419–433. 10.1007/s00248-017-1055-2 28875273

[B26] Gamfeldt L. Snall T. Bagchi R. Jonsson M. Gustafsson L. Kjellander P.. (2013). Higher levels of multiple ecosystem services are found in forests with more tree species. Nat. Commum. 4:1340. 10.1038/ncomms2328 23299890 PMC3562447

[B27] Gehring C. A. Sthultz C. M. Flores-Renteria L. Whipple A. V. Whitham T. G. (2017). Tree genetics defines fungal partner communities that may confer drought tolerance. Proc. Natl. Acad. Sci. U.S.A. 114, 11169–11174. 10.1073/pnas.1704022114 28973879 PMC5651740

[B28] Gherghel F. Behringer D. Haubrich S. Schlauss M. Fey-Wagner C. Rexer K.-H.. (2014). Former land use and host genotype influence the mycorrhizal colonization of poplar roots. Forests 5, 2980–2995. 10.3390/f5122980

[B29] Gloor G. B. Macklaim J. M. Pawlowsky-Glahn V. Egozcue J. J. (2017). Microbiome datasets are compositional: and this is not optional. Front. Microbiol. 8:2224. 10.3389/fmicb.2017.02224 29187837 PMC5695134

[B30] Haas J. C. Street N. R. Sjodin A. Lee N. M. Hogberg M. N. Nasholm T.. (2018). Microbial community response to growing season and plant nutrient optimisation in a boreal Norway spruce forest. Soil Biol. Biochem. 125, 197–209. 10.1016/j.soilbio.2018.07.005

[B31] Hoeber S. Arranz C. Nordh N. E. Baum C. Low M. Nock C.. (2018). Genotype identity has a more important influence than genotype diversity on shoot biomass productivity in willow short-rotation coppices. Glob. Change Biol. Bioenerg. 10, 534–547. 10.1111/gcbb.12521

[B32] Hoeber S. Fransson P. Weih M. Manzoni S. (2020). Leaf litter quality coupled to*Salix*variety drives litter decomposition more than stand diversity or climate. Plant Soil 453, 313–328. 10.1007/s11104-020-04606-0

[B33] Hrynkiewicz K. Baum C. Leinweber P. Weih M. Dimitriou I. (2010). The significance of rotation periods for mycorrhiza formation in short rotation coppice. For. Ecol. Manag. 260, 1943–1949. 10.1016/j.foreco.2010.08.020

[B34] Hrynkiewicz K. Toljander Y. K. Baum C. Fransson P. M. A. Taylor A. F. S. Weih M. (2012). Correspondence of ectomycorrhizal colonisation and diversity of willows (*Salix* spp.) in short rotation coppice on arable sites and adjacent natural stands. Mycorrhiza 22, 603–613. 10.1007/s00572-012-0437-z 22415721

[B35] Huson D. H. Mitra S. Ruscheweyh H.-J. Weber N. Schuster S. C. (2011). Integrative analysis of environmental sequences using MEGAN4. Genome Res. 21, 1552–1560. 10.1101/gr.120618.111 21690186 PMC3166839

[B36] Ihrmark K. Bodeker I. T. M. Cruz-Martinez K. Friberg H. Kubartova A. Schenck J.. (2012). New primers to amplify the fungal ITS2 region - evaluation by 454-sequencing of artificial and natural communities. FEMS Microbiol. Ecol. 82, 666–677. 10.1111/j.1574-6941.2012.01437.x 22738186

[B37] Kalucka I. L. Jagodzinski A. M. (2016). Successional traits of ectomycorrhizal fungi in forest reclamation after surface mining and agricultural disturbances: a review. Dendrobiology 76, 91–104. 10.12657/denbio.076.009

[B38] Karlinski L. Rudawska M. Leski T. (2013). The influence of host genotype and soil conditions on ectomycorrhizal community of poplar clones. Eur. J. Soil Biol. 58, 51–58. 10.1016/j.ejsobi.2013.05.007

[B39] Katanic M. Paoletti E. Orlovic S. Grebenc T. Kraigher H. (2014). Mycorrhizal status of an ozone-sensitive poplar clone treated with the antiozonant ethylene diurea. Eur. J. For. Res. 133, 735–743. 10.1007/s10342-013-0751-9

[B40] Koljalg U. Nilsson R. H. Abarenkov K. Tedersoo L. Taylor A. F. S. Bahram M.. (2013). Towards a unified paradigm for sequence-based identification of fungi. Mol. Ecol. 22, 5271–5277. 10.1111/mec.12481 24112409

[B41] Korkama T. Pakkanen A. Pennanen T. (2006). Ectomycorrhizal community structure varies among Norway spruce (*Picea abies*) clones. New Phytol. 171, 815–824. 10.1111/j.1469-8137.2006.01786.x 16918552

[B42] Kyaschenko J. Clemmensen K. E. Karltun E. Lindahl B. D. (2017). Below-ground organic matter accumulation along a boreal forest fertility gradient relates to guild interaction within fungal communities. Ecol. Lett. 20, 1546–1555. 10.1111/ele.12862 29057614

[B43] Lamit L. J. Holeski L. M. Flores-Renteria L. Whitham T. G. Gehring C. A. (2016). Tree genotype influences ectomycorrhizal fungal community structure: ecological and evolutionary implications. Fungal Ecol. 24, 124–134. 10.1016/j.funeco.2016.05.013

[B44] Lang C. Finkeldey R. Polle A. (2013). Spatial patterns of ectornycorrhizal assemblages in a monospecific forest in relation to host tree genotype. Front. Plant Sci. 4:103. 10.3389/fpls.2013.00103 23630537 PMC3633777

[B45] Lauber C. L. Strickland M. S. Bradford M. A. Fierer N. (2008). The influence of soil properties on the structure of bacterial and fungal communities across land-use types. Soil Biol. Biochem. 40, 2407–2415. 10.1016/j.soilbio.2008.05.021

[B46] Leake J. R. Read D. J. (2017). Mycorrhizal symbiosis and pedogenesis throughout Earth's history, in Mycorrhizal Mediation of Soil: Fertility, Structure and Carbon Storage, eds N. C. Johnson, C. Gehring, and J. Jansa (Amsterdam: Elsevier), 9–33. 10.1016/B978-0-12-804312-7.00002-4

[B47] Lilleskov E. A. Hobbie E. A. Horton T. R. (2011). Conservation of ectomycorrhizal fungi: exploring the linkages between functional and taxonomic responses to anthropogenic N deposition. Fungal Ecol. 4, 174–183. 10.1016/j.funeco.2010.09.008

[B48] Lodge D. J. Wentworth T. R. (1990). Negative associations among VA-mycorrhizal fungi and some ectomycorrhizal fungi inhabiting the same root-system. Oikos 57, 347–356. 10.2307/3565964

[B49] McCracken A. R. Walsh L. Moore P. J. Lynch M. Cowan P. Dawson M.. (2011). Yield of willow (*Salix* spp.) grown in short rotation coppice mixtures in a long-term trial. Ann. App. Biol. 159, 229–243. 10.1111/j.1744-7348.2011.00488.x

[B50] McCune B. (2006). PC-ORD. Multivariate Analysis of Ecological Data, 5.0 for Windows. Gleneden Beach, OR: MjM Software.

[B51] McCune B. Grace J. B. (2002). Analysis of Ecological Communities. Gleneden Beach, OR: MjM Software. pp. 304. 10.1016/S0022-0981(03)00091-1

[B52] Montgomery H. J. Monreal C. M. Young J. C. Seifert K. A. (2000). Determination of soil fungal biomass from soil ergosterol analyses. Soil Biol. Biochem. 32, 1207–1217. 10.1016/S0038-0717(00)00037-7

[B53] Nguyen N. H. Song Z. Bates S. T. Branco S. Tedersoo L. Menke J.. (2016). FUNGuild: An open annotation tool for parsing fungal community datasets by ecological guild. Fungal Ecol. 20, 241–248. 10.1016/j.funeco.2015.06.006

[B54] Nylund J. E. Wallander H. (1992). Ergosterol analysis as a means of quantifying mycorrhizal biomass. Methods Microbiol. 24, 77–88. 10.1016/S0580-9517(08)70088-6

[B55] Perez-Izquierdo L. Zabal-Aguirre M. Flores-Renteria D. Gonzalez-Martinez S. C. Buee M. Rincon A. (2017). Functional outcomes of fungal community shifts driven by tree genotype and spatial-temporal factors in Mediterranean pine forests. Environ. Microbiol. 19, 1639–1652. 10.1111/1462-2920.13690 28181376

[B56] Prescott C. E. Grayston S. J. (2013). Tree species influence on microbial communities in litter and soil: current knowledge and research needs. For. Ecol. Manag. 309, 19–27. 10.1016/j.foreco.2013.02.034

[B57] Püttsepp U. Rosling A. Taylor A. F. S. (2004). Ectomycorrhizal fungal communities associated with *Salix viminalis* L. *and S. dasyclados* Wimm. clones in a short-rotation forestry plantation. For. Ecol. Manag. 196, 413–424. 10.1016/j.foreco.2004.04.003

[B58] Quinn T. P. Erb I. Richardson M. F. Crowley T. M. (2018). Understanding sequencing data as compositions: an outlook and review. Bioinformatics 34, 2870–2878. 10.1093/bioinformatics/bty175 29608657 PMC6084572

[B59] R Core Team (2019). R: A Language and Environment for Statistical Computing. R Foundation for Statistical Computing, Vienna. Available online at: http://www.R-project.org/

[B60] Salmanowicz B. Nylund J. E. (1988). High-performance liquid-chromatography determination of ergosterol as a measure of ectomycorrhizal infection in Scots pine. Eur. J. For. Pathol. 18, 291–298. 10.1111/j.1439-0329.1988.tb00216.x

[B61] Scherer-Lorenzen M. Körner C. Schulze E.-D. (eds.). (2005). The functional significance of forest diversity: a synthesis, in Forest Diversity and Function: Temperate and Boreal Systems, (Berlin: Springer-Verlag), 377–389. 10.1007/3-540-26599-6_17

[B62] Schneider T. Keiblinger K. M. Schmid E. Sterflinger-Gleixner K. Ellersdorfer G. Roschitzki B.. (2012). Who is who in litter decomposition? Metaproteomics reveals major microbial players and their biogeochemical functions. ISME J. 6, 1749–1762. 10.1038/ismej.2012.11 22402400 PMC3498922

[B63] Schweitzer J. A. Bailey J. K. Fischer D. G. Leroy C. J. Lonsdorf E. V. Whitham T. G.. (2008). Plant-soil-microorganism interactions: heritable relationship between plant genotype and associated soil microorganisms. Ecology 89, 773–781. 10.1890/07-0337.1 18459340

[B64] Schweitzer J. A. Bailey J. K. Rehill B. J. Martinsen G. D. Hart S. C. Lindroth R. L.. (2004). Genetically based trait in a dominant tree affects ecosystem processes. Ecol. Lett. 7, 127–134. 10.1111/j.1461-0248.2003.00562.x

[B65] Schweitzer J. A. Fischer D. G. Rehill B. J. Wooley S. C. Woolbright S. A. Lindroth R. L.. (2011). Forest gene diversity is correlated with the composition and function of soil microbial communities. Popul. Ecol. 53, 35–46. 10.1007/s10144-010-0252-323236140

[B66] Shakya M. Gottel N. Castro H. Yang Z. M. K. Gunter L. Labbe J.. (2013). A multifactor analysis of fungal and bacterial community structure in the root microbiome of mature *Populus deltoides* trees. PLoS ONE 8:e76382. 10.1371/journal.pone.0076382 24146861 PMC3797799

[B67] Smith S. E. Read D. J. (eds.). (2008). Mycorrhizal Symbiosis. San Diego, CA: Academic Press.

[B68] Steinauer K. Chatzinotas A. Eisenhauer N. (2016). Root exudate cocktails: the link between plant diversity and soil microorganisms? Ecol. Evol. 6, 7387–7396. 10.1002/ece3.2454 28725406 PMC5513276

[B69] Tagu D. Bastien C. Faivre-Rampant P. Garbaye J. Vion P. Villar M.. (2005). Genetic analysis of phenotypic variation for ectomycorrhiza formation in an interspecific F1 poplar full-sib family. Mycorrhiza 15, 87–91. 10.1007/s00572-004-0302-9 15015061

[B70] Tedersoo L. Bahram M. Cajthaml T. Polme S. Hiiesalu I. Anslan S.. (2016). Tree diversity and species identity effects on soil fungi, protists and animals are context dependent. ISME J. 10, 346–362. 10.1038/ismej.2015.116 26172210 PMC4737927

[B71] Tedersoo L. Bahram M. Polme S. Koljalg U. Yorou N. S. Wijesundera R.. (2014). Global diversity and geography of soil fungi. Science 346, 1078–1089. 10.1126/science.1256688 25430773

[B72] Tedersoo L. Hansen K. Perry B. A. Kjoller R. (2006). Molecular and morphological diversity of pezizalean ectomycorrhiza. New Phytol. 170, 581–596. 10.1111/j.1469-8137.2006.01678.x 16626478

[B73] Tedersoo L. Sanchez-Ramirez S. Koljalg U. Bahram M. Doring M. Schigel D.. (2018). High-level classification of the Fungi and a tool for evolutionary ecological analyses. Fungal Divers. 90, 135–159. 10.1007/s13225-018-0401-0

[B74] van der Heijden M. G. A. Martin F. M. Selosse M.-A. Sanders I. R. (2015). Mycorrhizal ecology and evolution: the past, the present, and the future. New Phytol. 205, 1406–1423. 10.1111/nph.13288 25639293

[B75] van der Plas F. Manning P. Allan E. Scherer-Lorenzen M. Verheyen K. Wirth C.. (2016). Jack-of-all-trades effects drive biodiversity-ecosystem multifunctionality relationships in European forests. Nat. Commun. 7:11109. 10.1038/ncomms11109 27010076 PMC4820852

[B76] Velmala S. M. Rajala T. Haapanen M. Taylor A. F. S. Pennanen T. (2013). Genetic host-tree effects on the ectomycorrhizal community and root characteristics of Norway spruce. Mycorrhiza 23, 21–33. 10.1007/s00572-012-0446-y 22644394

[B77] Verheyen K. Vanhellemont M. Auge H. Baeten L. Baraloto C. Barsoum N.. (2016). Contributions of a global network of tree diversity experiments to sustainable forest plantations. Ambio 45:29–41. 10.1007/s13280-015-0685-1 26264716 PMC4709352

[B78] Voriskova J. Brabcova V. Cajthaml T. Baldrian P. (2014). Seasonal dynamics of fungal communities in a temperate oak forest soil. New Phytol. 201, 269–278. 10.1111/nph.12481 24010995

[B79] Weih M. (2004). Intensive short rotation forestry in boreal climates: present and future perspectives. Can. J. For. Res. 34, 1369–1378. 10.1139/x04-090

[B80] Weih M. Glynn C. Baum C. (2019). Willow short-rotation coppice as model system for exploring ecological theory on biodiversity–ecosystem function. Diversity 11:125. 10.3390/d11080125

[B81] Weih M. Nordh N.-E. Manzoni S. Hoeber S. (2021). Functional traits of individual varieties as determinants of growth and nitrogen use patterns in mixed stands of willow (*Salix* spp.). *For. Ecol. Manag*. 479:118605. 10.1016/j.foreco.2020.118605

[B82] Weih M. Nordh N. E. (2002). Characterising willows for biomass and phytoremediation: growth, nitrogen and water use of 14 willow clones under different irrigation and fertilisation regimes. Biomass Bioenerg. 23, 397–413. 10.1016/S0961-9534(02)00067-3

[B83] White T. J. Bruns T. Lee S. Taylor J. (1990). Amplification and direct sequencing of fungal ribosomal RNA genes for phylogenetics in PCR Protocols: A Guide to Methods and Applications, eds M. A. Innis, D. H. Gelfand, J. J. Sninsky, and T. J. White (San Diego, CA: Academic Press), 315–322. 10.1016/B978-0-12-372180-8.50042-1

[B84] Xue P-P. Carrillo Y. Pino V. Minasny B. McBratney A. B. (2018). Soil properties drive microbial community structure in a large scale transect in South Eastern Australia. Sci. Rep. 8:11725. 10.1038/s41598-018-30005-8 30082740 PMC6078944

