# Supplementary material for: Site-Dependent Relationships Between Fungal Community Composition, Plant Genotypic Diversity and Environmental Drivers in a Salix Biomass System
Source: Front Fungal Biol. 2021 Aug 13;2:671270. doi: 10.3389/ffunb.2021.671270 (PMC10512226; doi:10.3389/ffunb.2021.671270)
Supplement: Supplementary file 9 [file Data_Sheet_1.PDF]

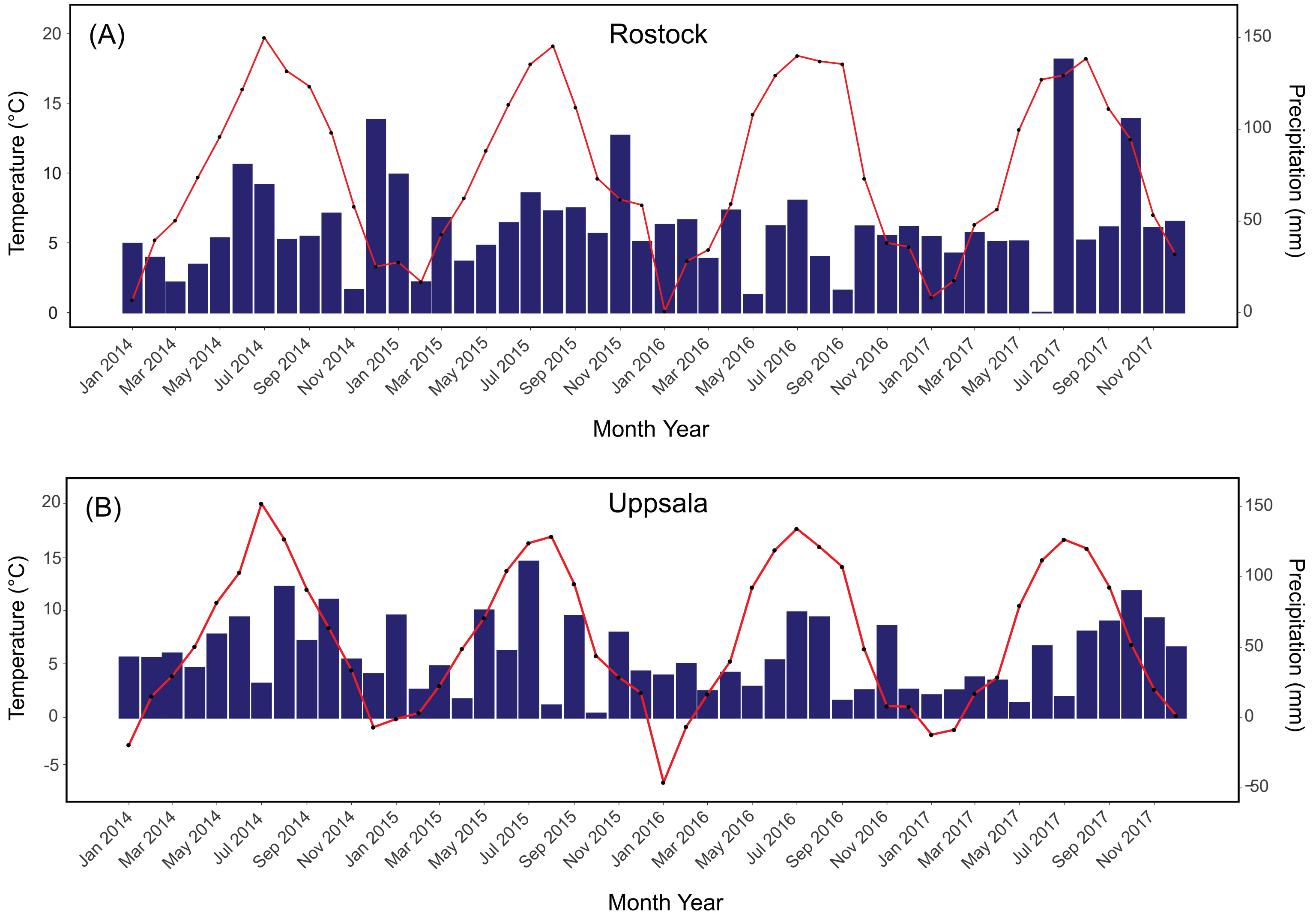

Supplementary Figure 1. Daily air temperature and monthly precipitation between 2014 and 2017 for (A) Rostock and (B) Uppsala.

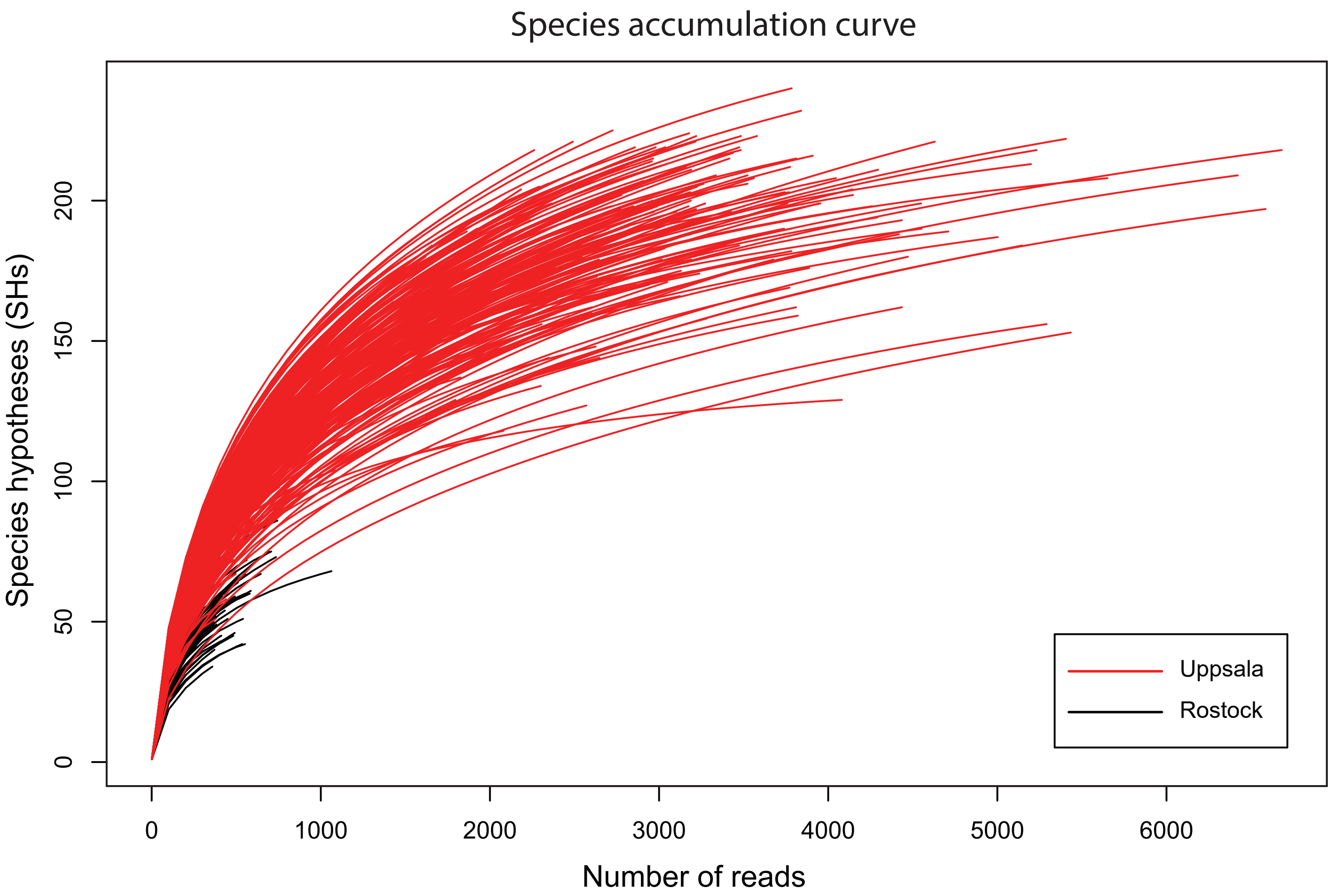

Supplementary Figure 2. Species accumulation curves for Rostock and Uppsala based on all study-level species hypothesis.

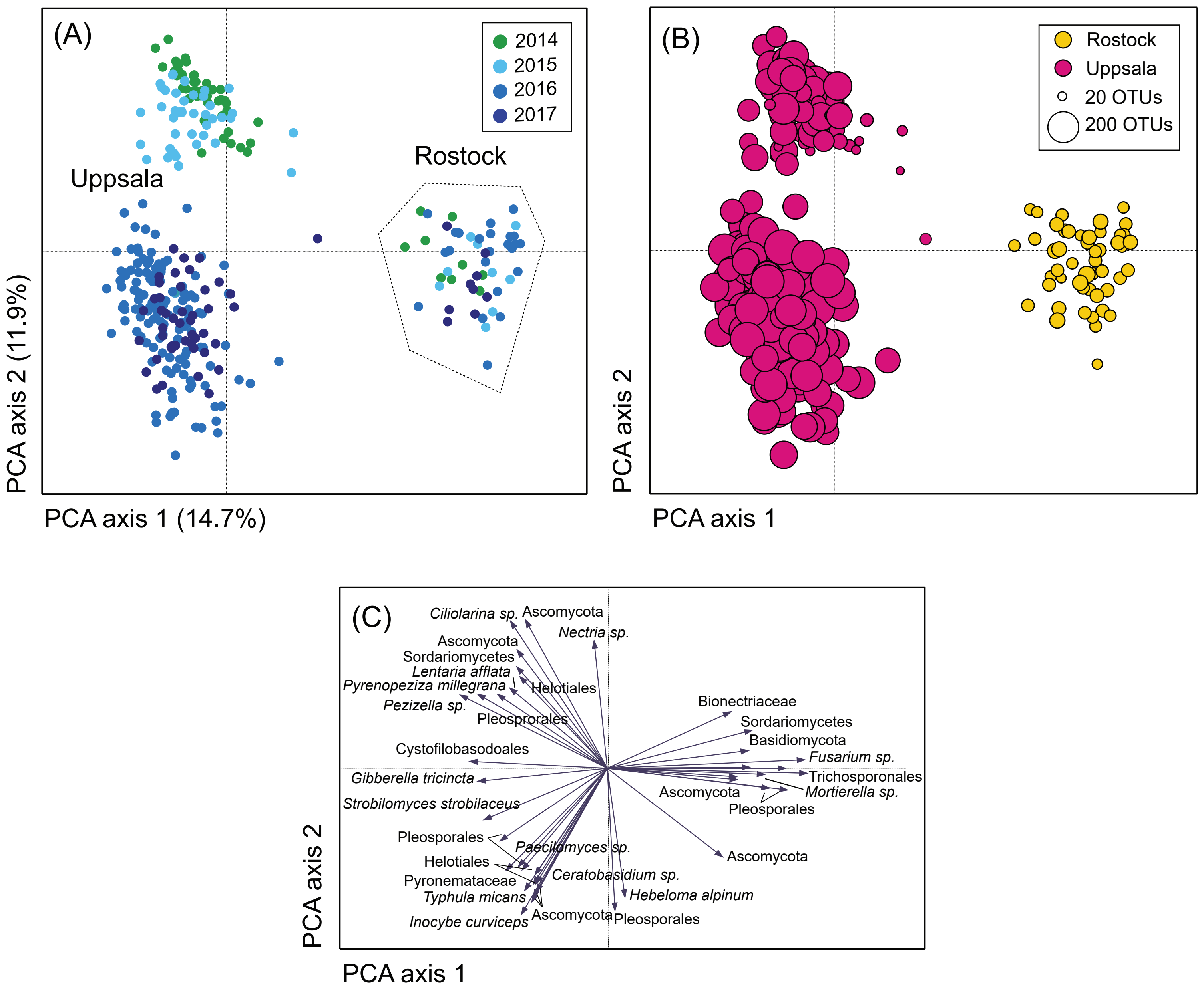

Supplementary Figure 3. Variation in soil fungal community composition in *Salix* genotype trials with the four genotypes (‘Björn’, ‘Jorr’, ‘Loden’ and ‘Tora’) planted in monoculture and various genotype mixtures (2-, 3- and 4-mixtures) at two different sites (Rostock, Germany and Uppsala, Sweden). Community composition is visualised by (A, B) a sample plot and (C) a species plot of a principle component analysis (PCA) based on PacBio sequencing of amplified ITS2 markers. The PCA was based on 446 identified fungal SHs, original data (not rarefied). Circles are colour coded according to: (A) year, and (B) site, and area indicating number of species hypothesis (SHs) in each sample. In (C) only the 40 most abundant SHs are shown. Axes 1 and 2 explained 14.7 and 11.9 %, respectively, of the total inertia of 1.2.

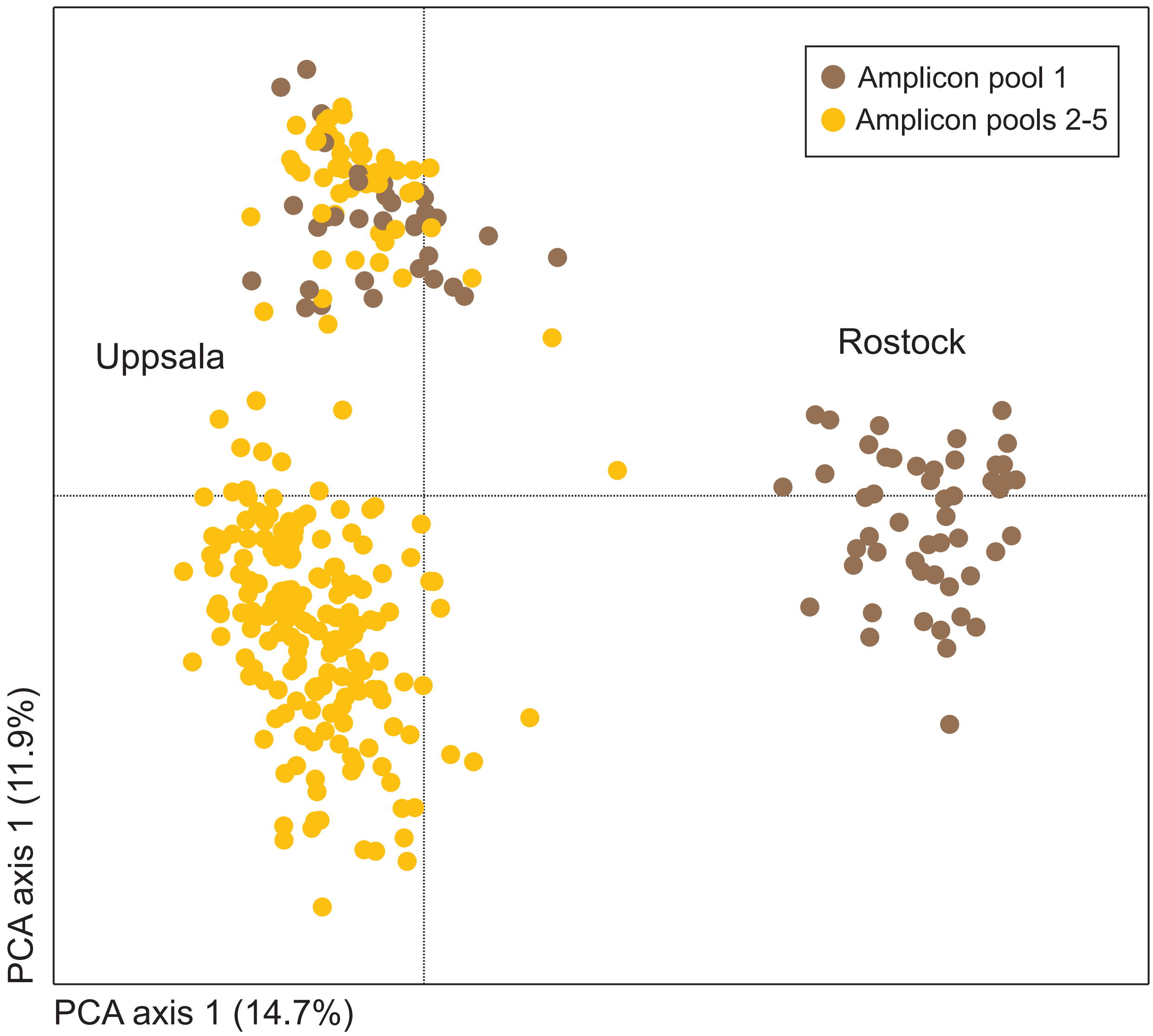

Supplementary Figure 4. Variation in soil fungal community composition in *Salix* genotype trials with the four genotypes ('Björn', 'Jorr', 'Loden' and 'Tora') planted in monoculture and various genotype mixtures (2-, 3- and 4-mixtures) at two different sites (Rostock, Germany and Uppsala, Sweden). Community composition is visualised by a sample plot of a principle component analysis (PCA) based on PacBio sequencing of amplified ITS2 markers. The PCA was based on 446 identified fungal SHs, original data (not rarefied). Circles are colour coded according to amplicon pool. Axes 1 and 2 explained 14.7 and 11.9 %, respectively, of the total inertia of 1.2. The plot is based on the same analysis as Figure S3. Although Uppsala samples in pool 1 were sequenced using a previous chemistry version compared to pools 2-5, community composition was very similar and samples from 2014 and 2015 grouped together irrespective of sequencing chemistry.

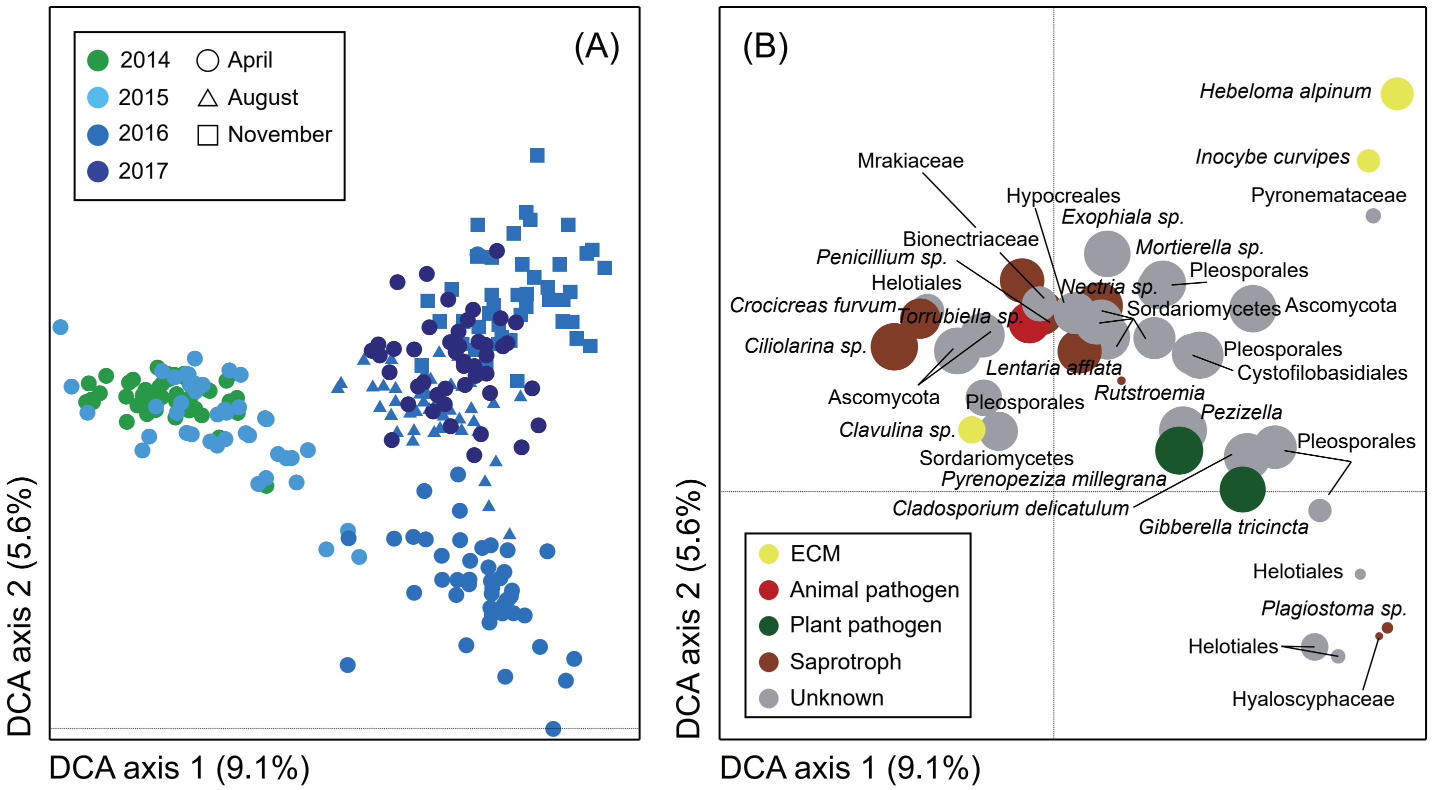

Supplementary Figure 5. Variation in soil fungal community composition in *Salix* genotype trial with four genotypes 'Björn', 'Jorr', 'Loden' and 'Tora' planted in monoculture and various genotype mixtures (2-, 3- and 4-mixtures) in Uppsala, Sweden. Community composition is visualised by (A) a sample plot and (B) a species plot of a detrended correspondence analysis (DCA), based on PacBio sequencing of amplified ITS2 markers, original data (not rarefied). The DCA was based on 446 identified fungal SHs. Symbols are colour coded according to: (A) year, with symbol shape indicating sampling month, and (B) functional groups with area indicating relative abundance. In the species plot (B) only the 40 most abundant SHs are shown. Axes 1 and 2 explained 9.1 and 5.6 %, respectively, of a total inertia of 2.4.

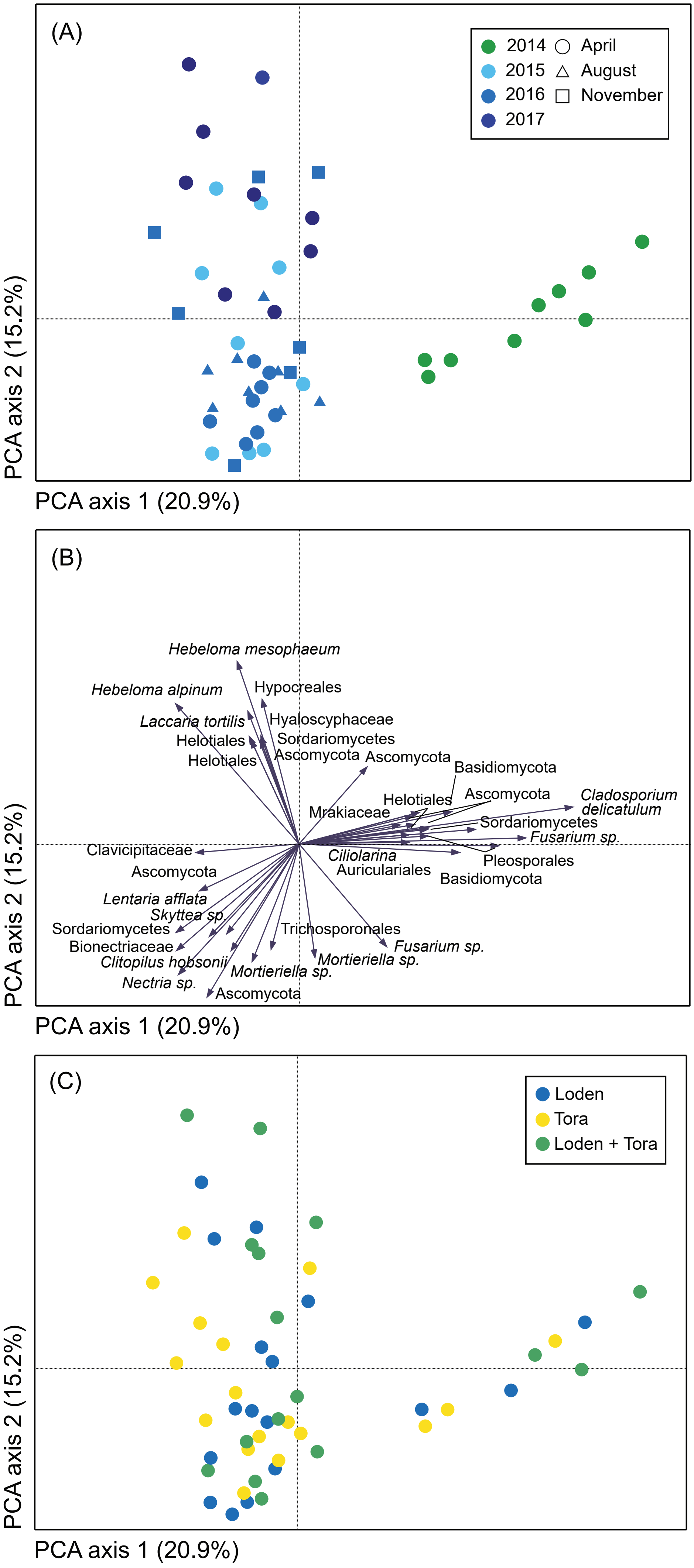
(A)
2014
2015
2016
2017
April
August
November
PCA axis 2 (15.2%)
PCA axis 1 (20.9%)
(B)
Hebeloma mesophaeum
Hebeloma alpinum
Hypocreales
Laccaria tortilis
Hyaloscyphaceae
Helotiales
Sordariomycetes
Helotiales
Ascomycota
Ascomycota
Basidiomycota
Helotiales
Ascomycota
Cladosporium delicatulum
Mrakiaceae
Sordariomycetes
Fusarium sp.
Clavicipitaceae
Ciliolarina
Auriculariales
Pleosporales
Basidiomycota
Ascomycota
Lentaria afflata
Skyttea sp
Sordariomycetes
Trichosporonales
Bionectriaceae
Fusarium sp.
Clitopilus hobsonii
Mortieriella sp.
Mortieriella sp.
Nectria sp.
Ascomycota
PCA axis 2 (15.2%)
PCA axis 1 (20.9%)
(C)
Loden
Tora
Loden + Tora
PCA axis 2 (15.2%)
PCA axis 1 (20.9%)

Supplementary Figure 6. Variation in soil fungal community composition in *Salix* genotype trial with two genotypes ('Loden' and 'Tora') planted in monocultures and 2-mixture in Rostock, Germany, visualised by (A, C) a sample plot and (B) a species plot of a principle component analysis (PCA) and based on PacBio sequencing of amplified ITS2 markers. The PCA was based on 245 identified fungal SHs, original data (not rarefied). Symbols are colour coded according to (A) year, with symbol shape indicating sampling month, and (C) genotype. Axes 1 and 2 explained 20.9 and 15.2 %, respectively, of a total inertia of 61.4. For the corresponding rarefied data set, the genotype coded PCA sample plot was similar (not shown).

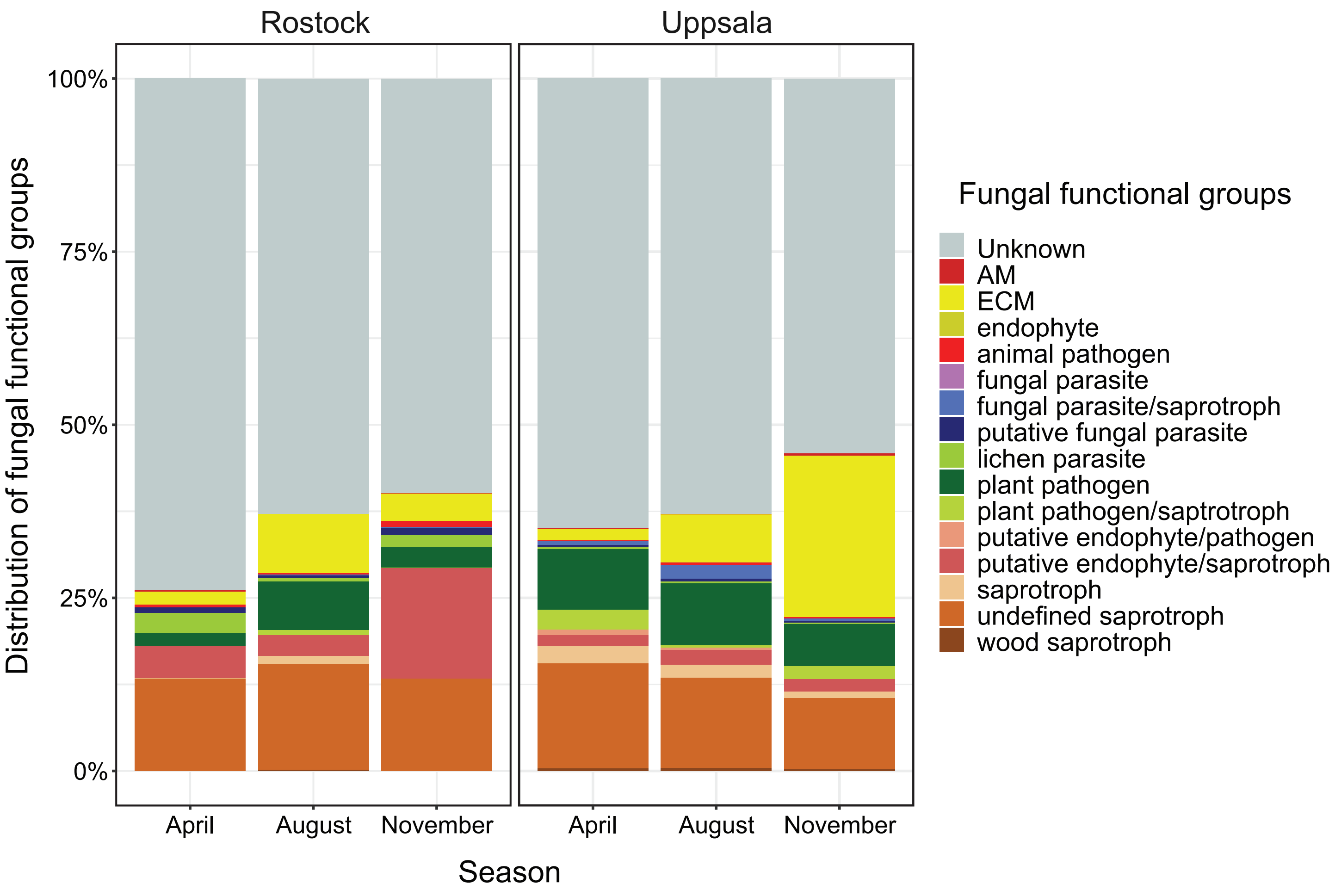

Supplementary Figure 7. Distribution of fungal functional groups in Rostock, Germany and Uppsala, Sweden over one growing season (April, August and November 2016), as estimated by PacBio sequencing of amplified ITS2 markers. Abundances are given as percent of the identified amplicon sequences, rarefied data (accounting for 80 % of total sequences).

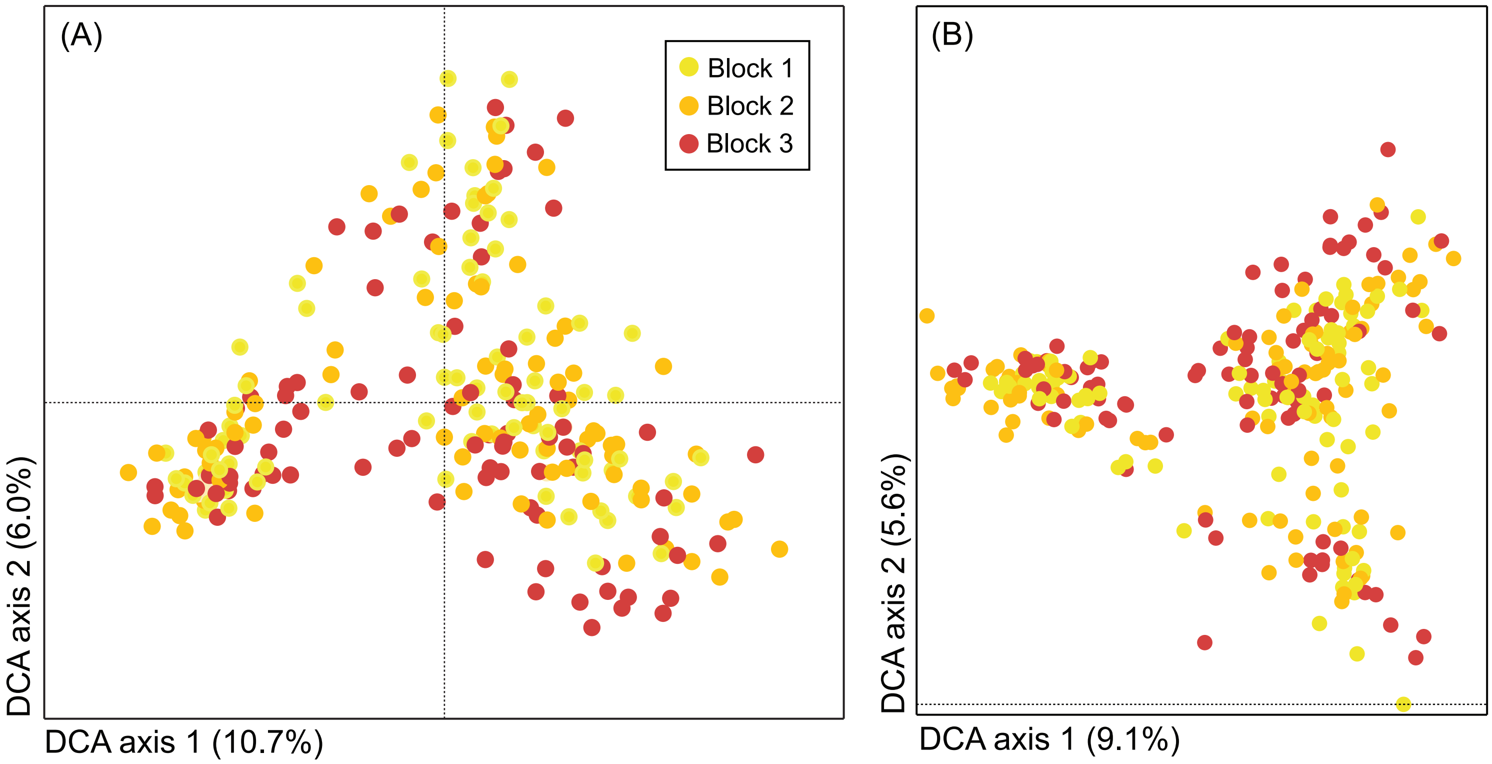

Supplementary Figure 8. Variation in soil fungal community composition in *Salix* genotype trials with the four genotypes ('Björn', 'Jorr', 'Loden' and 'Tora') planted in monoculture and various genotype mixtures (2-, 3- and 4-mixtures) in Uppsala, Sweden. Community composition is visualised by sample plots of detrended correspondence analyses (DCA), based on PacBio sequencing of amplified ITS2 markers. The DCA was based on (A) 442 identified fungal SHs, rarefied data, and (B) 446 identified fungal SHs, original data (not rarefied). Symbols are colour coded according to block. Axes 1 and 2 explained 9.1 and 5.6%, respectively, of a total inertia of 2.4. The plot is based on the same analyses as Figures 3 and S5 for (A) and (B), respectively.

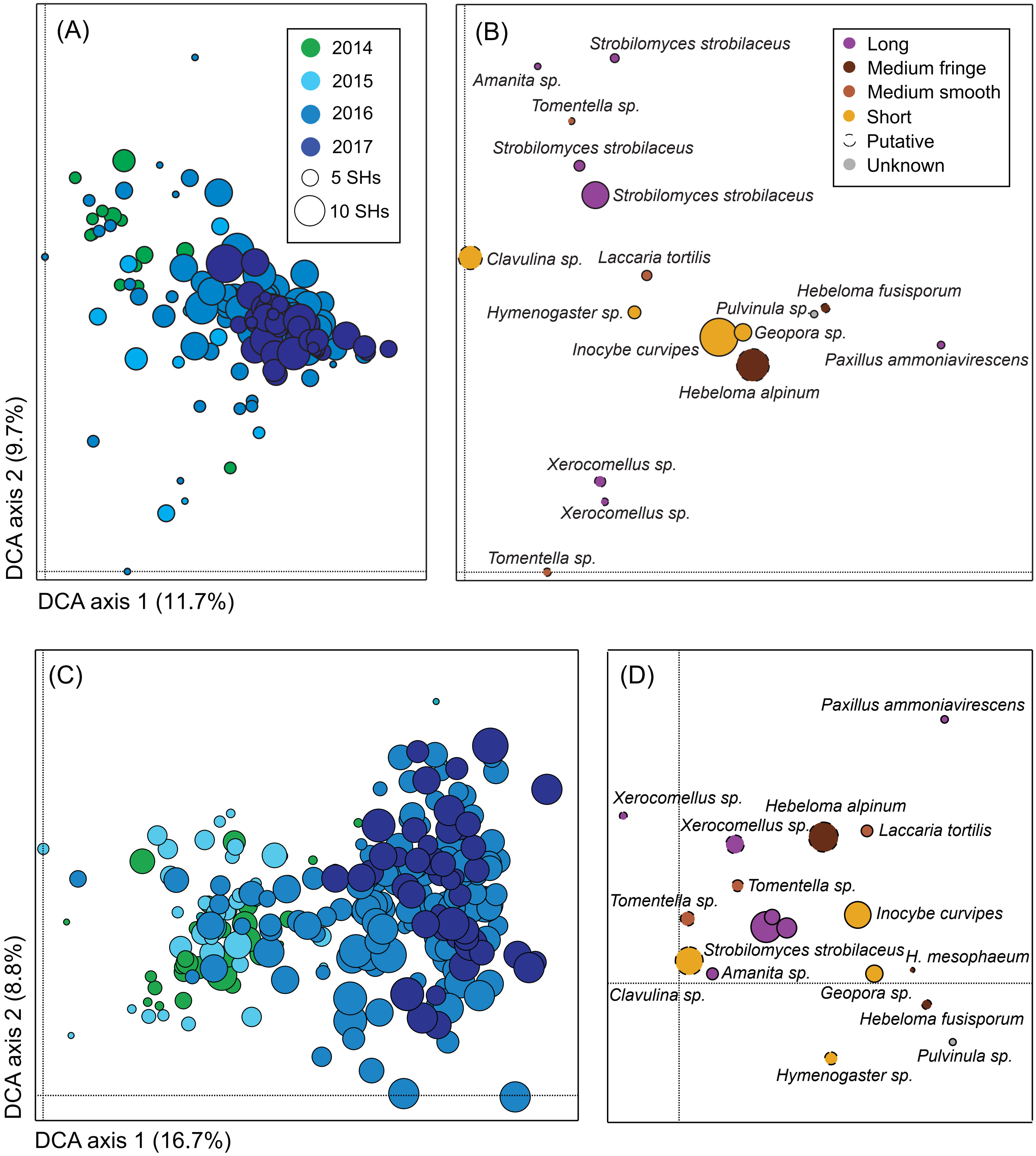

Supplementary Figure 9. Variation in ectomycorrhizal (ECM) fungal community composition in *Salix* genotype trial with four genotypes ('Björn', 'Jorr', 'Loden' and 'Tora') planted in monoculture and various genotype mixtures (2-, 3- and 4-mixtures) in Uppsala, Sweden. Community composition is visualised by (A, C) sample plots and (B, D) species plots of detrended correspondence analyses (DCA), based on PacBio sequencing of amplified ITS2 markers. The DCA was based on 18 ECM SHs; (A, B) shows rarefied data, and (C, D) original data (not rarefied). Symbols are colour coded according to (A, C) year and (B, D) exploration types, with area indicating total number of taxa in each sample. Axes 1 and 2 explained 11.7 and 9.7 %, respectively, of a total inertia of 5.4 for the rarefied data, and 16.7 and 8.8 % of a total inertia of 2.0 for the original data (not rarefied).
